# Immunophenotyping TCF1-expressing TILs: spatial profiling and prognostic value in operable non-small cell lung cancer

**DOI:** 10.3389/fimmu.2026.1731337

**Published:** 2026-01-22

**Authors:** Konstantinos Ntostoglou, Georgios Christodoulopoulos, Katie Stoker, Jean Descarpentrie, Anastasia Xagara, Dora Chatzidaki, Vasiliki Anastasopoulou, Ilias P. Nikas, Argyro Ioanna Ieronimaki, Lucy Booth, Sophia Tsoka, Ioannis Vamvakaris, Apostolos Klinakis, Eleni Patsea, Sophia N. Karagiannis, Ioannis G. Panayiotides, Teresa Frisan, Vassilis Georgoulias, Athanasios Kotsakis, Ioannis S. Pateras

**Affiliations:** 1 Hellenic Oncology Research Group (HORG), Athens, Greece; 2 Laboratory of Oncology, Faculty of Medicine, School of Health Sciences, University of Thessaly, Larissa, Greece; 3 Department of Medical Oncology, University General Hospital of Larissa, Larissa, Greece; 4 St. John’s Institute of Dermatology, School of Basic and Medical Biosciences and KHP Centre for Translational Medicine, King’s College London, Guy’s Hospital, London, United Kingdom; 5 Breast Cancer Now Research Unit, School of Cancer and Pharmaceutical Sciences, King’s College London, Guy’s Cancer Centre, London, United Kingdom; 6 Department of Informatics, Faculty of Natural, Mathematical and Engineering Sciences, King’s College London, London, United Kingdom; 7 Department of Molecular Biology and Umeå Centre for Microbial Research (UCMR), Umeå University, Umeå, Sweden; 8 Biomedical Research Foundation of the Academy of Athens, Athens, Greece; 9 Medical School, University of Cyprus, Nicosia, Cyprus; 10 2^nd^ Department of Pathology, “Attikon” University Hospital, Medical School, National and Kapodistrian University of Athens, Athens, Greece; 11 Department of Pathology, ‘Sotiria’ General Hospital for Chest Diseases, Athens, Greece; 12 Department of Pathology, Metropolitan Hospital, Athens, Greece

**Keywords:** biomarkers, non-small cell lung cancer, spatial analysis, T cell factor 1, tumor infiltrating lymphocytes, tumor microenvironment

## Abstract

**Background:**

The spatial distribution and functional heterogeneity of tumor-infiltrating lymphocytes (TILs) significantly impact patient outcomes in non-small cell lung cancer (NSCLC). While T cell factor 1 (TCF1) expressing TILs have emerged as key players in sustaining anti-tumor immunity, their subset characterization, localization, and clinical significance within the tumor microenvironment remain poorly defined.

**Method:**

We performed multiplex immunohistochemistry and immunofluorescence to characterize TCF1^+^ immune cell subsets, in 102 NSCLC tumors, separately analyzing the tumor center (TC) and invasive front (IF). We integrated this data with publicly available single-cell RNA-sequencing datasets and clinical outcome analyses.

**Results:**

CD4^+^ T cells and CD79α^+^ B cells, dominate the TCF1^+^ landscape, while CD8^+^ T cells constitute a minority of TCF1^+^ immune cells, particularly in the TC. We demonstrated the presence of tumor-infiltrating IgG^+^/IgA^+^ plasma cells co-expressing TCF1. PD1^+^TCF1^-^ cells were more frequent than PD1^+^TCF1^+^ cells both in the TC and IF, reflecting that terminally differentiated exhausted TILs predominate within the tumor microenvironment. Survival analyses revealed significantly different prognostic impact of TILs including TCF1-expressing cells based on topography. Multivariate analysis showed that increased CD8^+^TCF1^+^ cells (HR: 2.5; p=0.039) and increased TCF1 expression by cancer cells (HR: 2,7; p=0.041) in the TC and CD4^+^TCF1^+^ cells (HR: 0.4; p=0.043) in the IF emerged as negative and positive independent prognostic markers for Disease-free survival (DFS), respectively. Integrating PD-L1 expression with TILs, PD-L1 immunopositivity was correlated with increased CD8^+^ and PD1^+^TCF1^-^ cell infiltration and was associated with favorable DFS especially in the TC.

**Conclusions:**

Our findings support a more refined framework for TCF1^+^ TIL assessment and TCF1 expression across cellular populations in the tumor microenvironment, with implications for prognostication in operable NSCLC.

## Introduction

Non-small cell lung cancer (NSCLC) remains one of the most common malignancies and a leading cause of cancer-associated death throughout the world (1). In early stage and locally advanced NSCLC, surgical resection is the treatment of choice. However, 30-55% of patients develop postoperative recurrence, indicating the importance of developing appropriate prognostic biomarkers (2). Accumulating evidence demonstrates the clinical value of the tumor microenvironment (TME), including tumor-infiltrating lymphocytes (TILs) in NSCLC (3–5).

CD8^+^ TILs have gained attention as they constitute the main anti-cancer effector cells of the adaptive immune response. However, in the TME, T cells often acquire a state of T cell exhaustion, which describes the compromised function of immune cells (6). Recent advances identified progenitor (early) exhausted T cells with stem cell- or memory-like features that express T cell factor 1 (TCF1, encoded by *TCF7*) along with low to moderate levels of inhibitory signals including the checkpoint molecule programmed death 1 (PD1), and terminally differentiated (late) exhausted T cells which lack TCF1 expression, but are characterized by high PD1 expression (6, 7). Expression of TCF1 has been linked to the maintenance of T cell effector functions, while loss of TCF1, along with concomitant up-regulation of inhibitory molecules, is associated with a decline in effector functions (8). The clinical relevance of stem cell-like CD8^+^TCF1^+^ cells was initially demonstrated in melanoma patients, showing that patients with high CD8^+^TCF1^+^ cell count showed improved response to immune-checkpoint inhibitors (ICIs) (9). Similar findings were observed in NSCLC and invasive breast carcinoma (10–12). Besides, we recently demonstrated the strong prognostic value of CD8^+^TCF1^+^ cells in triple-negative breast cancer, and Huang et al. showed similar findings for colorectal cancer (13, 14). However, TCF1-expression across immune cell subsets, localization, and clinical significance of TCF1^+^ cells in the tumor microenvironment (TME) remain poorly defined.

Tumor-infiltrating B lymphocytes comprise a critical component of TILs (15). However, relative to T cells, the role of B lymphocytes in cancer is understudied. Several studies demonstrate that increased infiltration of TME by CD20^+^ B cells has been reported to be associated with favorable prognosis in breast cancer, melanoma and NSCLC (16). As B cells are central players of humoral immunity, knowledge of B cell biology in NSCLC could improve our understanding of the TME and help us to develop new biomarkers and therapeutic strategies.

The current study aims to dissect the immunophenotypic profile along with the clinical value of TCF1-expressing cells in the TME of tumors from a well-characterized cohort of 102 treatment-naïve NSCLC patients with early and locally advanced stage. We investigated which cells express TCF1, and whether their distribution differs between the tumor center (TC) and the invasive front (IF) along with their prognostic value.

## Materials and methods

### Study population

This prospective translational research study, conducted by the Hellenic Oncology Research Group (HORG), enrolled 102 treatment-naïve patients (during the period 2018-2023) with histologically documented stage I-III (according to IASLC/UICC, 8^th^ edition) NSCLC that underwent surgical resection based on the Multidisciplinary Tumor Board’s (MTD) decision. Forty-six (45.1%) patients were diagnosed with squamous cell carcinoma (SCC), 53 (48.0%) with adenocarcinomas (ADC) whereas 7 (6.9%) patients were grouped as “other” histological types including patients with pleomorphic carcinoma (n=3) and large cell carcinoma (n=1) (**Supplementary Table 1**).

### *In situ* analysis

For *in situ* analysis of immune infiltrate we performed single and double immunohistochemistry (IHC) as well as multiplex immunofluorescence on formalin-fixed paraffin-embedded (FFPE) tissue sections obtained from the resected primary tumor. For the *in situ* analysis we assessed each marker separately in the TC and the IF as previously described (17).

#### (i) Immunohistochemistry

For single and double IHC the following antibodies were employed: anti-CD8 (clone C8/144B, 108 M, Cell Marque, 1:60); anti-CD4 (clone 4B12, IS649, DAKO, ready to use), anti-CD79α (JCB117, DAKO, ready to use), anti-TCF1 (clone C63D9, 2203, Cell Signaling, 1:50), anti-PD1 (NAT105, Cell Marque, 1:50) and anti-PDL1 (clone SP263, Roche). PD-L1 immunostaining was performed on Ventana Benchmark Ultra staining system (Roche Diagnostics, Tucson, AZ, USA). Sections were counterstained with Mayer’s hematoxylin. To exclude non-specific staining in double immunostainings, we omitted sequentially the primary antibody. Human tonsil served as a positive control for CD8, CD4, CD79α, TCF1 and PD-L1. For CD8, CD4, CD79α and TCF1 we assessed the number of positive (+) stromal cells per high-power field (HPF, magnification 400x) as previously described (13, 17), as well as separately the percentage of TCF1 (%) expressed by cancer cells. For the evaluation of PD-L1, we employed the Tumor Proportion Score (TPS). PD-L1 immunopositivity was defined as TPS score ≥1. The assessment, of CD8^+^TCF1^+^, CD4^+^TCF1^+^, CD79α^+^TCF1^+^, PD1^+^TCF1^+^ and PD1^+^/TCF1^-^ stromal cells was performed by counting the number of (+) cells per HPF.

#### (ii) Multiplex immunofluorescence

For multiplex immunofluorescence we employed a panel of five markers comprising the following primary antibodies: anti-CD8 (108 M, Cell Marque, 1:60); anti-CD4 (ab133616 Abcam, 1:100), anti-CD79α (ab79414, Abcam, 1:100), anti-TCF1 (2203, Cell Signaling, 1:50), and anti-Pancytokeratin (KRT/1877R, ab234297, 1:100). The following secondaries antibodies were employed: goat-anti-mouse IgG (H+L) Alexa Fluor™ 488 (A11029) and goat-anti-rabbit IgG (H+L) Alexa Fluor™ 568 (A11011) purchased from Thermo Fisher. We performed sequential rounds of staining, each including goat serum and 5% BSA blocking, followed by primary antibody and matched secondary antibody. To remove primary and secondary antibodies (stripping) across rounds of staining we employed Lunaphore LabSat^®^ using Elution buffer Solution 2 (20X) (REF BU07, Lunaphore technologies) diluted in Solution 1 (1X) (REF BU07, Lunaphore technologies). Nuclei were counterstained with DAPI/VECTASHIELD^®^ (H-1200-100, Vector Laboratories). Stained sections were scanned, and images were acquired using the 3D HISTECH Pannoramic MIDI (Pannoramic software 4.1.0). Tonsils were used as a positive control. To exclude non-specific staining, we omitted the primary antibodies.

### Bioinformatic analysis

#### Single-cell RNA sequencing data processing

Publicly available NSCLC single-cell RNA sequencing raw counts data was downloaded from GSE127465, GSE131907, GSE136246 and GSE153935 (18–21). Single-cell data was analyzed in R (v4.3.0) using the Seurat (v5.1.0) package. Raw counts matrix and associated metadata were loaded into R. The Seurat object containing 135, 376 cells across 42 patient samples was generated using the CreateSeuratObject() function including a minimum of 200 features, which are detected in a minimum of 3 cells. The object was normalized and scaled using the NormalizeData() and ScaleData() functions respectively. The FindNeighbors() function was used to construct a nearest-neighbor graph using the top 9 principal components (PCs). Clustering was performed with FindClusters() using a resolution of 0.5. Clusters were visualized on a reduced-dimensionality Uniform Manifold Approximation and Projection (UMAP) plot.

#### Batch correction

Clusters were visualized per study accession ID revealing batch effects in the data (**Supplementary Figures S1A, B**). Batch correction was performed using the Seurat integrative analysis method, whereby the Seurat object was normalized by SCTransform() and integrated using the IntegrateLayers() function using RPCAIntegration (**Supplementary Figures S2A–D**). Clustering was performed with FindClusters() using a resolution of 0.5. Clusters were visualized on a reduced-dimensionality UMAP plot (**Supplementary Figures S3A–C**).

#### Cell type annotation and re-clustering

The Seurat object was first prepared for differential gene expression analysis using the PrepSCTFindMarkers() function. Differential genes were calculated using the FindAllMarkers() function with, testing genes that are detected in a minimum 20% cells. DEGs were filtered to include only significant genes (padj ≤ 0.05). A combination of top significant DEGs and a panel of key cell markers were used for annotation. Immune cells including B, T and plasma cells were selected for re-clustering to annotate these populations to a higher resolution. The FindNeighbors() function was used to construct a nearest-neighbor graph using the top 9 principal components (PCs). Clustering was performed with FindClusters() using a resolution of 0.3. Seurat functions including VlnPlot() and FeaturePlot() were used to compare *TCF7* expression between cell types.

### Statistical analysis

Due to the descriptive nature of the present study, no formal statistical hypothesis was tested for sample size estimation.

Summary tables (descriptive statistics and/or frequency tables) were provided for all baseline and efficacy variables, as appropriate. Continuous variables were summarized with descriptive statistics (n, median and range). Qualitative factors were compared using Pearson’s chi-square test or Fisher’s exact test, whenever appropriate. For each biomarker, the Mann-Whitney’s U test was used to compare the two groups of each tested variable. The biomarker values were assessed graphically using boxplots displaying both the medians and the ranks of each subtype.

Disease-free survival (DFS) was defined as the time of NSCLC initial diagnosis until the date of first documented disease relapse. To evaluate the impact of each biomarker expression on the DFS, Kaplan–Meier (KM) curves in association with univariate Cox regression analyses were performed with computation of a hazard ratio and 95% confidence interval. In the KM analysis, comparisons were conducted using the log-rank test. Multivariate analysis was performed on variables with a p-value of less than 0.05 in the univariate analysis. Median follow-up was calculated using the reverse Kaplan-Meier method. All statistical tests were two-sided, and p-values <0.05 were considered statistically significant.

The X-tile algorithm (20) was used to determine the optimal cut-off value for each biomarker with patients being stratified into high- and low-expression groups (**Supplementary Material**) (22). X-tile graphs are also provided as **Supplementary Material**. The data were analyzed using the IBM SPSS statistics for Windows (IBM Corp. Released 2019. IBM SPSS Statistics for Windows, Version 26.0. Armonk, NY: IBM Corp) predictive analysis software.

## Results

### Spatial profiling and distinct prognostic impact of infiltrating CD8^+^TCF1^+^ T cells

**Figure 1:**
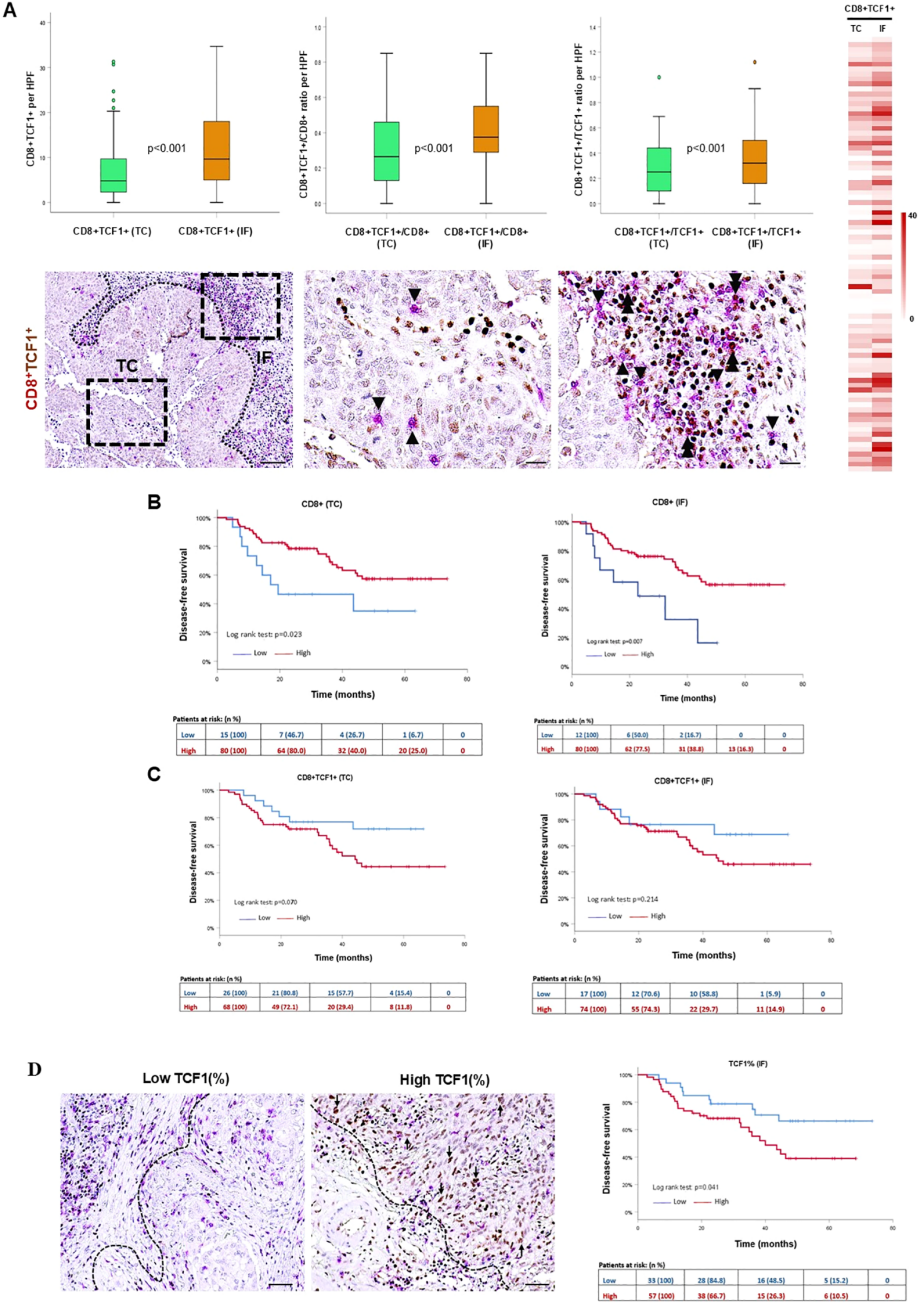
Spatial distribution and prognostic impact of CD8^+^TCF1^+^ T cells in the tumor center (TC) and invasive front (IF). **(A)** Upper panel: quantification of CD8^+^TCF1^+^ cells, and CD8^+^TCF1^+^/CD8^+^ and CD8^+^TCF1^+^/TCF1^+^ ratios between TC and IF. Lower panel: representative micrographs showing CD8^+^ (red) and TCF1^+^ (brown) cells. Arrowheads depict CD8^+^ T cells, and double arrowheads demonstrate CD8^+^TCF1^+^ cells. Scale bar: 100μm (low magnification); 20μm (high magnification) HPF: high power field. Heat map analysis of CD8^+^TCF1^+^ cells in the TC and the IF **(B, C)** Kaplan-Meier survival curves of CD8^+^ and CD8^+^TCF1^+^ cell infiltration in the TC and IF. **(D)** Left panel: representative micrograph of TCF1 staining (brown) in cancer cells (arrows). Scale bar: 100μm. Right panel: Kaplan-Meier survival curves of TCF1 expression (evaluated a percentage positive cells) by cancer cells in the IF.

Double immunohistochemistry analysis was used to assess the presence and distribution of CD8^+^TCF1^+^ T lymphocytes in the TC and the IF. Both the absolute number of CD8^+^TCF1^+^ T cells as well as the CD8^+^TCF1^+^/CD8^+^ and CD8^+^TCF1^+^/TCF1^+^ ratios, were higher in the IF compared to the TC (**Figure 1A**), mirroring the distribution observed for CD8^+^ T cells (**Supplementary Figure S4**).

We next examined the prognostic impact of CD8^+^ and CD8^+^TCF1^+^ T cells separately in the TC and the IF. The presence of CD8^+^ cells both in the TC and IF was associated with a prolonged DFS (TC: HR:0.4, 95%CI:0.2-0.9, p=0.027; IF: HR:0.4, 95%CI:0.2-0.8, p=0.010, respectively). Unexpectedly, high levels of CD8^+^TCF1^+^ cells revealed a statistical trend towards worse DFS in the TC (**Figures 1B, C**, **Table 1**). Likewise, increased CD8^+^TCF1^+^/CD8^+^ and CD8^+^TCF1^+^/TCF1^+^ ratios in the TC were associated with worse DFS (HR: 2.9, 95%CI:1.5-5.9, p=0.002; HR: 2.9, 95%CI:1.2-7.0, p=0.018) (**Table 1**).

**Table 1:** KM & Univariate Cox regression analysis for DFS, evaluating the prognostic role of CD8/TCF1 and CD4/TCF1 status separately in the tumor center (TC) and invasive front (IF).

CD8+	DFS – TC				DFS – IF			
	KM analysis		Univ. cox regression		KM analysis		Univ. cox regression	
	Median (range)	p-value	HR (95%C.I)	p-value	Median (range)	p-value	HR (95%C.I)	p-value
Low (ref)	19.4 (4.9–63.2)	**0.023**	0.4 (0.2-0.9)	**0.027**	22.8 (4.9-50.3)	**0.007**	0.4 (0.2-0.8)	**0.010**
High	NR (2.9 – 73.5)				NR (2.9-73.5)			
TCF1+								
Low (ref)	NR (2.9 – 73.5)	0.198	1.6 (0.8-3.2)	0.202	NR (2.9 – 73.5)	0.065	1.9 (0.9-3.7)	0.069
High	NR (6.8 – 67.7)				35.8 (6.8-61.7)			
TCF1+ (%) *								
Low (ref)	NR (6.6 – 73.5)	0.047	2.5 (1.0-6.5)	0.055	NR (6.6 – 73.5)	0.041	2.1 (1.0-4.4)	**0.045**
High	NR (2.9 – 68.4)				NR (2.9 – 68.4)			
CD8+TCF1+								
Low (ref)	NR (7.8 – 66.5)	0.070	2.1 (0.9-4.8)	0.076	NR (6.6 – 66.5)	0.214	1.8 (0.7-4.7)	0.221
High	NR (2.9 – 73.5)				NR (2.9 – 73.5)			
CD8+TCF1+/CD8+								
Low (ref)	NR (2.9 – 73.5)	0.001	2.9 (1.5-5.9)	**0.002**	NR (6.6 – 73.5)	0.074	2.0 (0.9-4.4)	0.080
High	21.7 (4.9-61.8)				38.2 (2.9-61.7)			
CD8+TCF1+/TCF1+								
Low (ref)	NR (7.8 – 67.7)	0.013	2.9 (1.2-7.0)	**0.018**	NR (4.9 – 73.5)	0.126	0.5 (0.2-1.2)	0.134
High	NR (2.9 – 73.5)				NR (2.9 – 68.4)			
CD4+								
Low (ref)	NR (2.9 – 68.3)	0.056	2.3 (0.9-5.7)	0.063	31.9 (2.9 – 61.8)	0.004	0.3 (0.2-0.7)	**0.005**
High	14 (6.6 – 55.9)				NR (6.6 – 68.3)			
CD4+TCF1+								
Low (ref)	NR (2.9 – 68.3)	0.193	1.7 (0.7-4.0)	0.198	35.9 (2.9 – 61.7)	0.013	0.4 (0.2-0.8)	**0.016**
High	44.8 (6.6 – 61.7)				NR (6.6 – 68.3)			
CD4+TCF1+/TCF1+								
Low (ref)	NR (2.9 – 67.7)	0.367	0.7 (0.4-1.5)	0.370	NR (2.9 – 67.7)	0.272	0.7 (0.3-1.4)	0.276
High	NR (4.9 – 68.3)				NR (4.9 – 68.3)			
CD4+TCF1+/CD4+								
Low (ref)	NR (4.9 – 67.7)	0.298	0.7 (0.3-1.4)	0.301	NR (2.9 – 66.5)	0.298	0.7 (0.3-1.4)	0.301
High	NR (2.9 – 68.3)				NR (4.9 – 68.3)			

NR, not reached; TCF1+(%) * refers to TCF1-expression by cancer cells.

Significant *P* values (< 0.05) linked with the biomarkers’ expression are written in bold.

As TCF1 was also expressed by cancer cells, we assessed its prognostic impact and found that increased percentage of TCF1^+^ in cancer cells was associated with a strong trend for a worse DFS in the TC (HR:2.5, 95%CI:1.0-6.5 p=0.055), a trend that became significant when the TCF1 positivity was assessed in the IF (HR:2.1, 95%CI:1.0-4.4, p=0.045) (**Table 1**, **Figure 1D**).

### TCF1-expressing cells in the TME of NSCLC patients

The immunohistochemical analysis demonstrated that the majority of TCF1^+^ immune cells did not co-express CD8 in TME (**Figure 1A**). Therefore, we characterized the different TCF1^+^ subpopulations in NSCLC by performing bioinformatics analysis based on publicly available NSCLC single-cell RNA sequencing data (18–21).

**Figure 2:**
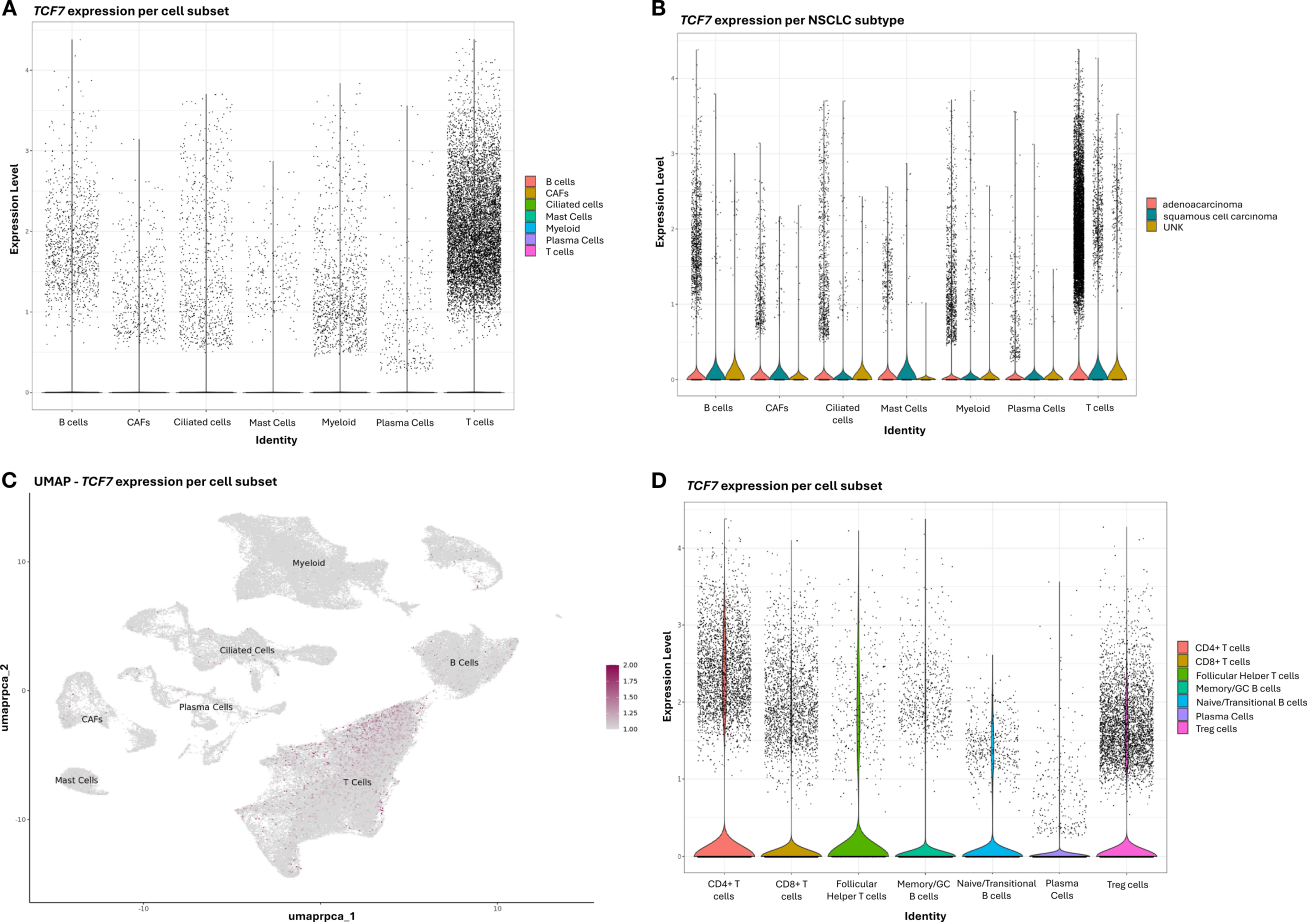
**(A–D)** Single-cell RNA sequencing analysis of *TCF7* expression across NSCLC. **(A, B)** Violin plot shows *TCF7* expression across the annotated cell types from NSCLC patient samples and separately across different NSCLC subtypes UNK: unknown NSCLC subtype. **(C)** Feature plot shows *TCF7* expression across the cell type clusters on a UMAP. **(D)** Feature plot shows *TCF7* expression across T and B subtypes in NSCLC.

This analysis identified several subsets of immune cells and non-immune cells expressing TCF1 mRNA (*TCF7*) in NSCLC (**Figures 2A–C**). As expected, higher expression levels of *TCF7* were found in T cells, independently of NSCLC subtypes (**Figure 2B**). Interestingly, a smaller proportion of myeloid and mast cells also expressed *TCF7* (**Figures 2A–D**). In addition, non-immune populations such as cancer associated fibroblasts (CAFs) and ciliated cells were identified as *TCF7*-expressing cells (**Figures 2A–C**).

Within the adaptive immunity population, *TCF7* was expressed in CD8^+^ and CD4^+^ cells, including CD4^+^ subsets such as T regulatory (Treg) and follicular helper cells (**Figure 2D**), as well as in B cell subsets, including naïve/transitional, germinal center/memory B cells, and plasma cells (**Figure 2D**).

**Figure 3:**
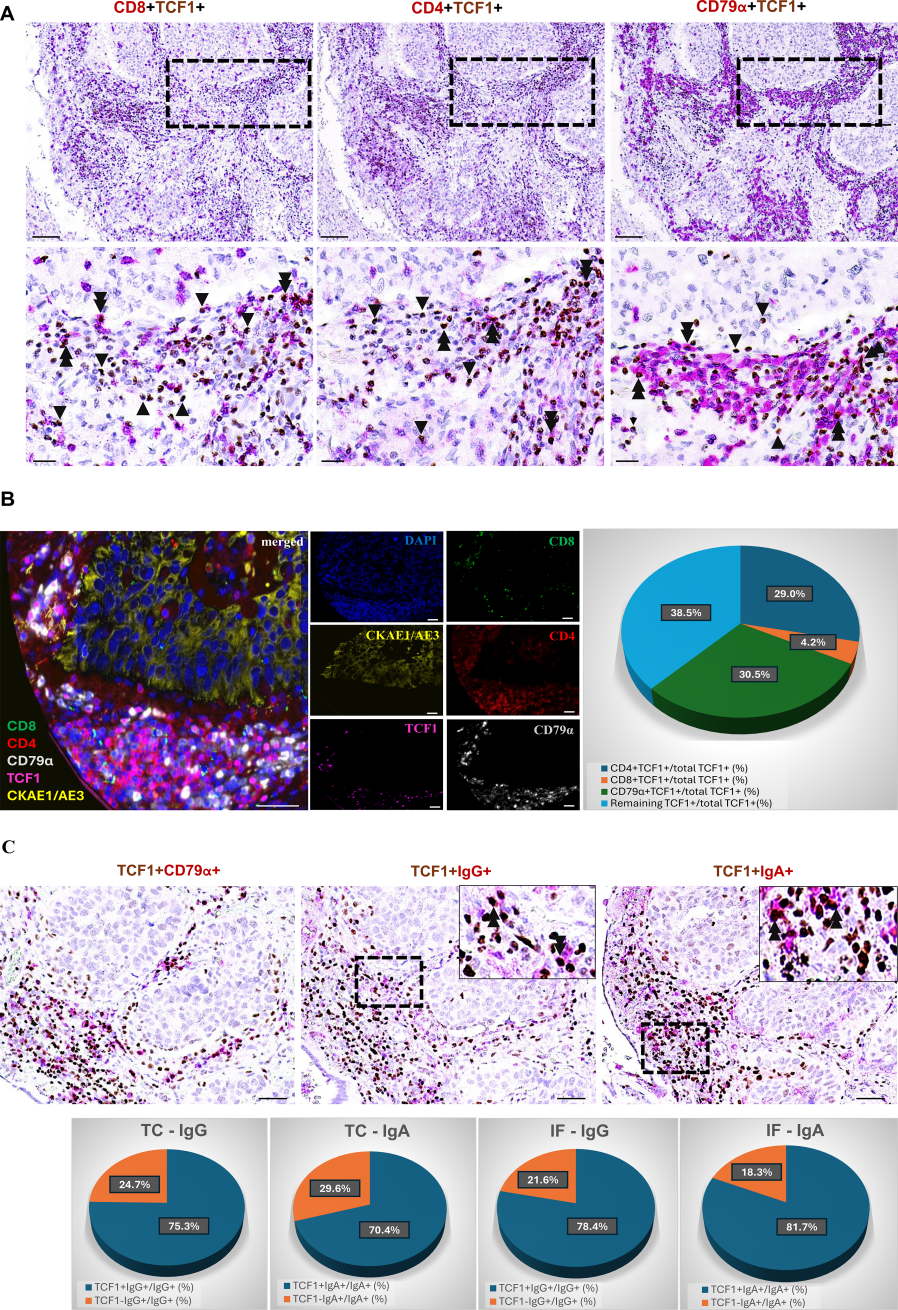
*In situ* assessment of TCF1 expression by T and B cells. **(A)** Serial section of double immunohistochemistry analysis for CD8^+^TCF1^+^, CD4^+^TCF1^+^ and CD79α^+^TCF1^+^ cell infiltrate. Scale bar: 100μm (high magnification); 20μm (low magnification). **(B)** Left panel: representative micrograph of the multiplex immunofluorescence staining for CD8 (green), CD4 (red), CD79α (grey) and TCF1 (pink) on the same section. Epithelial cells were stained with CKAE1/AE3 (yellow). Nuclei were counterstained with DAPI (blue). Scale bar: 100μm; 20μm (insert). Right panel: quantification of the percentage of CD8^+^TCF1^+^/TCF1^+^, CD4^+^TCF1^+^/TCF1^+^ and CD79α^+^TCF1^+^/TCF1^+^ in the TC. **(C)** Upper panel: representative micrograph of the serial section analysis of TCF1/CD79α, ΤCF1/IgG and TCF1/IgA cell infiltrate. Scale bar: 50μm. Lower panel: quantification of the percentage of TCF1^+^IgG^+^/IgG^+^ and TCF1^+^IgA^+^/IgA^+^ in the TC and the IF.

Prompted by these findings, we performed double immunohistochemistry in serial tissue sections to assess the presence of CD8^+^TCF1^+^ and CD4^+^TCF1^+^ T cells, as well as CD79α^+^TCF1^+^ B cells in TME at protein level (**Figure 3A**). To quantify the percentage of CD8^+^TCF1^+^, CD4^+^TCF1^+^ and CD79α^+^TCF1^+^ cells on the same sections a multiplex immunofluorescence analysis was performed in a patient subset, revealing, that 30.5% of TCF1-expressing cells in the TC are CD79α^+^, followed by CD4^+^ (29.0%) and to a lesser degree CD8^+^ (4.2%) (**Figure 3B**) confirming the immunohistochemistry data presented in **Figure 3A**. Interestingly, 38.5% of TCF1^+^ cells were negative for all three markers.

Among the CD79α^+^TCF1^+^ cells, many exhibited an eccentric nucleus, a key morphological feature of plasma cells, validating the bio-informatic analysis (**Figure 3A**). As CD79α is a pan-B cell marker expressed throughout B cell differentiation, including the plasma cell stage (23), IgG and IgA were used as markers of plasma cells (24). The majority of IgG^+^ and IgA^+^ plasma cells co-expressed TCF1 both in the TC and the IF in a subset of 10 patients (**Figure 3C**).

Prompted by our findings, we focused on CD4^+^TCF1^+^ and CD79α^+^TCF1^+^ cells, assessing their distribution separately in the TC and IF along with their prognostic impact.

### Spatial profiling and distinct prognostic impact of infiltrating CD4^+^TCF1^+^ T cells

**Figure 4:**
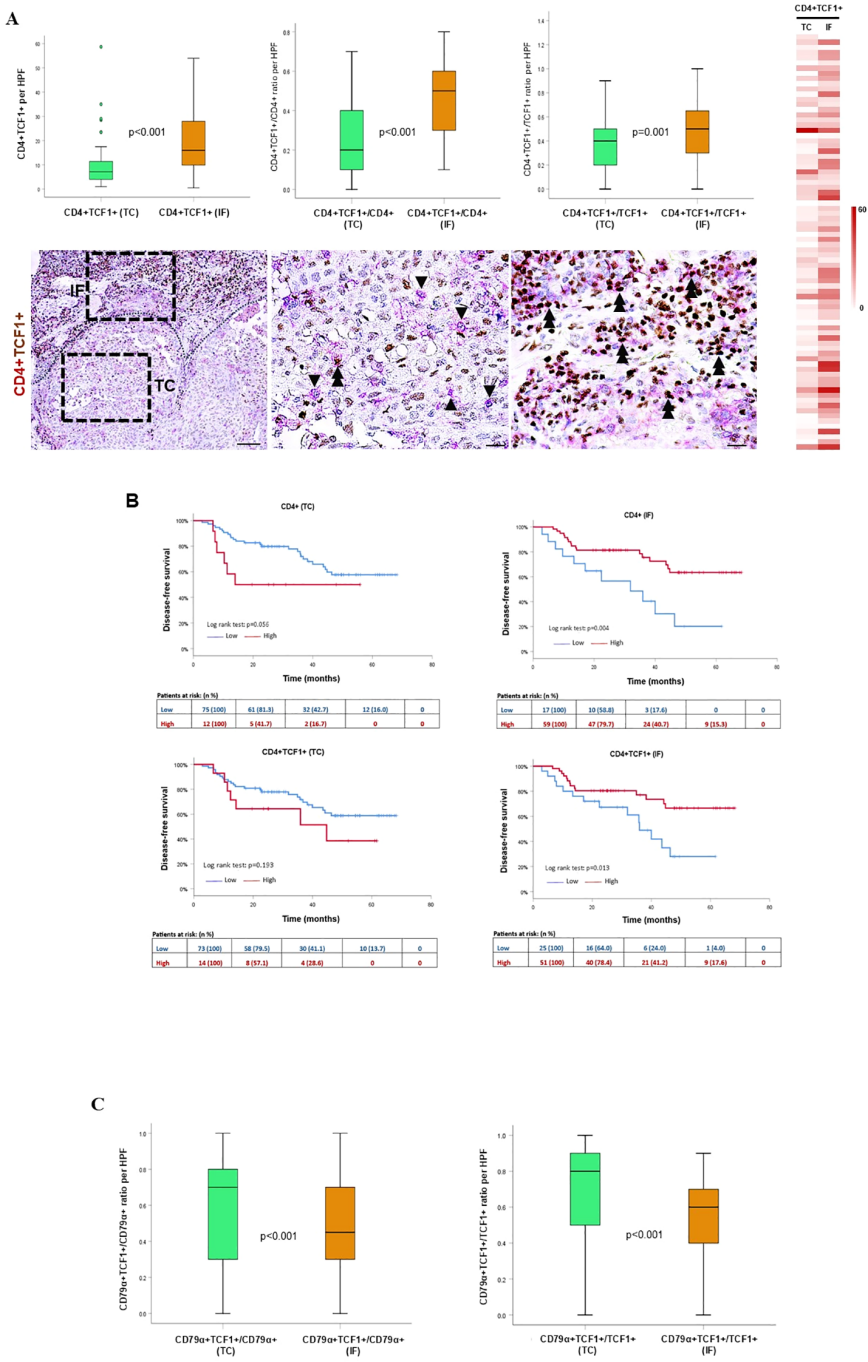
Spatial distribution and prognostic impact of CD4^+^TCF1^+^ T cells and CD79α^+^TCF1^+^ B cells in the tumor center (TC) and invasive front (IF). **(A)** Upper panel: quantification of CD4^+^TCF1^+^ cells, and CD4^+^TCF1^+^/CD4^+^ and CD4^+^TCF1^+^/TCF1^+^ ratios between TC and IF. Lower panel: representative micrographs showing CD4^+^ (red) and TCF1^+^ (brown) cells. Arrowheads depict CD4^+^ T cells, and double arrowheads indicate CD4^+^TCF1^+^ cells. Scale bar: 100μm (low magnification); 20μm (high magnification) HPF: high power field. Heat map analysis of CD4^+^TCF1^+^ cells in the TC and the IF. **(B)** Kaplan-Meier survival curves of CD4^+^ and CD4^+^TCF1^+^ cell infiltration in the TC and IF. **(C)** Quantification of CD79α^+^TCF1^+^/CD79α^+^ and CD79α^+^TCF1^+^/TCF1^+^ ratios in the TC and IF.

Spatial profiling revealed that both the absolute number of CD4^+^TCF1^+^ T cells as well as the CD4^+^TCF1^+^/CD4^+^ and CD4^+^TCF1^+^/TCF1^+^ ratios were significantly enriched in the IF compared to the TC (**Figure 4A**), in accordance with higher CD4^+^ T cell density (**Supplementary Figure S5**) and mirroring the pattern observed for CD8^+^ T cells (**Supplementary Figure S4**). Interestingly, increased CD4^+^ cell density in the TC revealed a trend associated with worse DFS (HR:2.3, 95%CI:0.9-5.7, p=0.063), whereas it was significantly linked with favorable DFS in the IF (HR:0.3, 95%CI:0.2-0.7, p=0.005) (**Figure 4B**, **Table 1**). Similarly, only increased CD4^+^TCF1^+^ T cell density in the IF but not in the TC significantly correlated with improved DFS (HR:0.4, 95%CI:0.2-0.8, p=0.016), (**Figure 4B**, **Table 1**).

Collectively, these data show that, unlike CD8^+^TCF1^+^ cells, the presence of CD4^+^TCF1^+^ cells in the IF is associated with a favorable prognosis, suggesting distinct roles for these two cell types within the TME, and highlight a compartment-specific prognostic role for CD4^+^TCF1^+^ T cells in NSCLC.

### Spatial profiling and distinct prognostic impact of infiltrating CD79α^+^TCF1^+^ B cells

The CD79α^+^TCF1^+^/CD79α^+^ and CD79α^+^TCF1^+^/TCF1^+^ ratios were significantly higher in the TC compared to the IF (**Figure 4C**), showing an opposite pattern observed for CD8^+^ and CD4^+^ T cells. We did not find any significant differences in the absolute number of CD79α^+^ and CD79α^+^TCF1^+^ cells between the TC and the IF (**Supplementary Figure S6**).

Survival analysis demonstrated that high CD79α+ cell count in the TC but not in the IF was associated with a trend towards favorable DFS [HR: 0.4, 95%CI: 0.2-1.0, p=0.058] (**Supplementary Table 2**). No significant associations were found between CD79α^+^TCF1^+^ cells with DFS irrespective of the tumor area (**Supplementary Table 2**).

### Spatial profiling of progenitor versus terminally differentiated exhausted TILs

**Figure 5:**
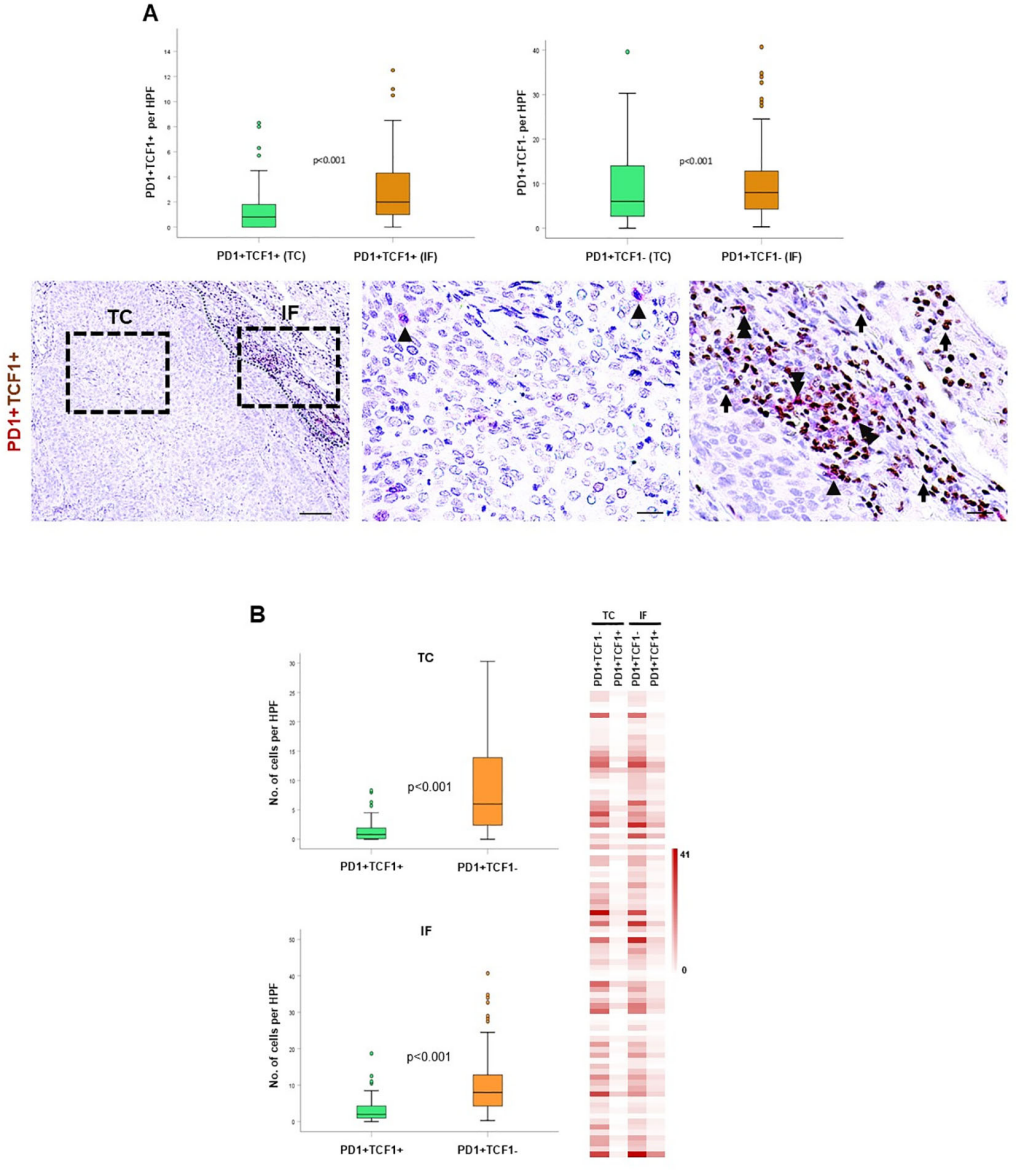
Spatial distribution and prognostic impact of PD1^+^TCF1^+^ and PD1^+^TCF1^-^ cells in the tumor center (TC) and invasive front (IF). **(A)** Upper panel: quantification of PD1^+^TCF1^+^ and PD1^+^TCF1^-^ cells in the TC and IF. Lower panel: representative micrographs showing PD1^+^ (red) and TCF1^+^ (brown) cells. Arrowheads depict PD1^+^ cells, and double arrowheads show PD1^+^TCF1^+^ cells. Scale bar: 100μm (low magnification); 20μm (high magnification), HPF: high power field. **(B)** Quantification of PD1^+^TCF1^+^ and PD1^+^TCF1^-^ cells in the TC and the IF. HPF: high power field. Heat map analysis of PD1^+^TCF1^+^ and PD1^+^TCF1^-^ cells in the TC and the IF.

The co-expression of TCF1 with PD-1 is considered a marker for exhausted lymphocytes. Thus, we evaluated the spatial distribution of exhausted TILs by distinguishing between PD-1^+^TCF1^+^ progenitor-like exhausted cells and PD-1^+^TCF1^-^ terminally differentiated exhausted cells. Both populations were significantly enriched in the IF compared to TC (**Figure 5A**). Interestingly, PD-1^+^TCF1^-^ cells were significantly higher than PD-1^+^TCF1^+^ cells (**Figure 5B**), suggesting a higher percentage of TILs expressing a terminally exhausted phenotype in the tumor area. Survival analysis could not reveal any significant association between the density of PD-1^+^ TCF1^+^ and PD-1^+^ TCF1^-^ cells with DFS (**Supplementary Table 3**).

### Increased PD-L1 status is associated with favorable survival

**Figure 6:**
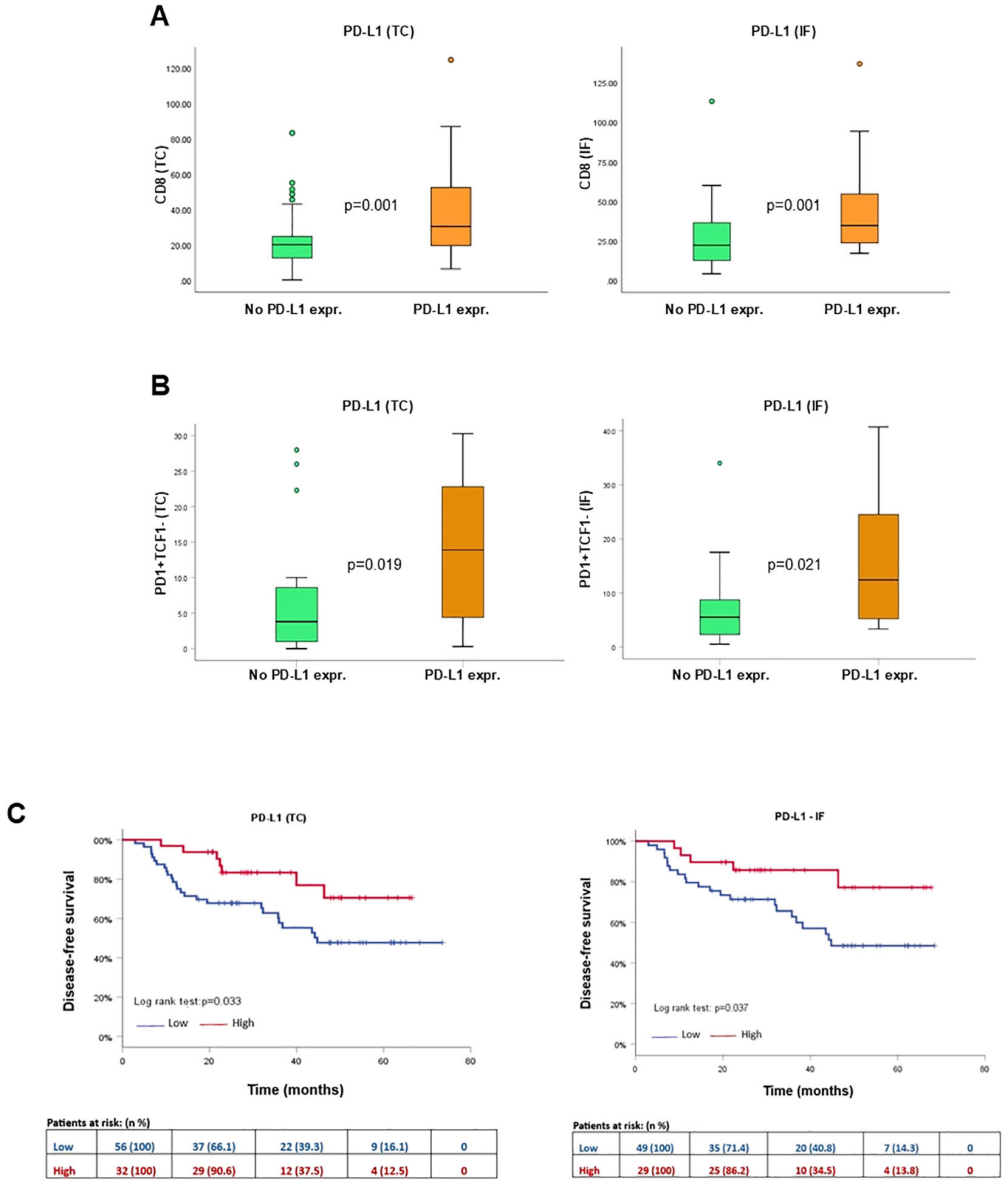
Spatial distribution and prognostic impact of PD-L1 status in the tumor center (TC) and invasive front (IF). Quantification of CD8^+^**(A)** and CD8^+^TCF1^+^**(B)** cell distribution in cases with PD-L1 TPS<1% (No PD-L1) versus cases with PD-L1 TPS score ≥1% (PD-L1 expr) in the TC and IF. **(C)** Kaplan-Meier survival curve of PD-L1 status in the TC and the IF.

As Programmed Death Ligand 1 (PD-L1) is the ligand of PD1, we assessed the PD-L1 status in the NSCLC cohort. PD-L1 immunopositivity was found in the TC and IF in 36.4% and 37.2% of NSCLC cases, respectively. Integrating PD-L1 with CD8^+^ and CD8^+^TCF1^+^ status, PD-L1 immunopositivity was significantly associated with increased CD8^+^ T cell density both in the TC and in IF, as well as with increased CD8^+^TCF1^+^ cell count only in the IF (**Figure 6A**, **Supplementary Table 4**). As expected, PD-L1 immunopositivity was associated with increased PD1^+^TCF1^-^ cell count in both the TC and the IF, reflecting an increase in terminally exhausted TILs (**Figure 6B**, **Supplementary Table 5**).

Patients with tumors harboring PD-L1 TPS ≥1% were significantly associated with favorable DFS both in the TC (HR:0.4, 95%CI:0.2-0.9, p=0.039) and the IF (HR:0.4, 95%CI:0.1-0.9, p=0.045) (**Figure 6C**, **Supplementary Table 6**).

### Multivariate analysis

Multivariate analysis adjusted for tumor stage, sex and age (26), revealed that increased CD8^+^ (HR = 0.4, 95%CI: 0.2-0.8, p=0.017) cells and high PD-L1 (HR:0.4, 95%CI: 0.2 – 1.0, p=0.041) status in the TC are independent prognostic markers linked with favorable DFS (**Table 2**). Furthermore multivariate analysis showed that increased CD8^+^TCF1^+^ (HR:2.5, 95%CI: 1.1 – 6.2, p=0.039) cell density, CD8^+^TCF1^+^/CD8^+^ (HR: 2.7, 95%CI: 1.3 – 5.4, p=0.007) and CD8^+^TCF1^+^/TCF1^+^ (HR: 3.1, 95%CI: 1.3 – 7.6, p=0.011) ratios as well as increased TCF1(%) (HR: 2.7, 95%CI: 1.1 – 7.0, p=0.041) status in the TC emerged as independent significant prognostic factors associated with worse DFS (**Table 2**). In addition, the multivariate analysis demonstrated an independent significant association of increased CD8^+^ (HR: 0.3, 95%CI: 0.1 – 0.7, p=0.005), CD4^+^ (HR: 0.3, 95%CI: 0.1 – 0.7, p=0.005) and CD4^+^TCF1^+^ (HR: 0.4, 95%CI: 0.2 – 0.9, p=0.043) cell density in the IF with improved DFS (**Table 3**).

**Table 2:** Multivariate Cox regression analysis for DFS, evaluating the prognostic impact of each biomarker (separate model for each biomarker, with adjustments made each time for the following covariates: stage, age and sex) in the tumor center (TC).

Biomarker	Variable	HR (95% C.I)	p-value
CD8+	CD8+ [L (ref) vs H]	0.4 (0.2 – 0.8)	**0.017**
	Stage [I (ref) vs II + III]	2.6 (1.3 – 5.3)	0.008
	Sex [M (ref) vs F]	0.8 (0.3 – 1.7)	0.495
	Age [≥65 (ref) vs <65]	1.4 (0.6 – 3.1)	0.399
TCF1+%	TCF1+% [L (ref) vs H]	2.7 (1.1 – 7.0)	**0.041**
	Stage [I (ref) vs II + III]	2.5 (1.2 – 5.0)	0.012
	Sex [M (ref) vs F]	0.8 (0.4 – 1.6)	0.513
	Age [≥65 (ref) vs <65]	1.1 (0.5 – 2.4)	0.717
CD8+TCF1+	CD8+TCF1+ [L (ref) vs H]	2.5 (1.1 – 6.2)	**0.039**
	Stage [I (ref) vs II + III]	2.4 (1.2 – 4.7)	0.015
	Sex [M (ref) vs F]	0.7 (0.3 – 1.6)	0.440
	Age [≥65 (ref) vs <65]	1.1 (0.5 – 2.4)	0.721
CD8+TCF1+/CD8+	CD8+TCF1+/CD8+ [L (ref) vs H]	2.7 (1.3 – 5.4)	**0.007**
	Stage [I (ref) vs II + III]	2.1 (1.1 – 4.3)	0.039
	Sex [M (ref) vs F]	0.8 (0.4 – 1.8)	0.585
	Age [≥65 (ref) vs <65]	1.3 (0.5 – 2.8)	0.555
CD8+TCF1+/TCF1+	CD8+TCF1+/TCF1+ [L (ref) vs H]	3.1 (1.3 – 7.6)	**0.011**
	Stage [I (ref) vs II + III]	2.4 (1.2 – 4.8)	0.017
	Sex [M (ref) vs F]	0.7 (0.3 – 1.6)	0.411
	Age [≥65 (ref) vs <65]	1.2 (0.5 – 2.5)	0.684
CD4+	CD4+ [L (ref) vs H]	4.0 (1.5 – 10.7)	**0.007**
	Stage [I (ref) vs II + III]	3.2 (1.5 – 7.1)	0.003
	Sex [M (ref) vs F]	0.8 (0.4 – 1.9)	0.656
	Age [≥65 (ref) vs <65]	1.9 (0.8 – 4.3)	0.117
CD79α+	CD79α+ [L (ref) vs H]	0.4 (0.2 – 1.0)	0.062
	Stage [I (ref) vs II + III]	2.6 (1.3 – 5.3)	0.009
	Sex [M (ref) vs F]	0.8 (0.4 – 1.8)	0.669
	Age [≥65 (ref) vs <65]	1.1 (0.5 – 2.5)	0.780
PD-L1	PD-L1 [No expr. (ref) vs Expr.]	0.4 (0.2 – 1.0)	**0.041**
	Stage [I (ref) vs II + III]	2.5 (1.2 – 5.1)	0.017
	Sex [M (ref) vs F]	0.6 (0.3 – 1.4)	0.262
	Age [≥65 (ref) vs <65]	1.5 (0.7 – 3.5)	0.290

Significant *P* values (< 0.05) linked with the biomarkers’ expression are written in bold.

**Table 3:** Multivariate Cox regression analysis for DFS, evaluating the prognostic impact of each biomarker (separate model for each biomarker, with adjustments made each time for the following covariates: stage, age and sex) in the invasive front (IF).

Biomarker	Variable	HR (95% C.I)	p-value
CD8+	CD8+ [L (ref) vs H]	0.3 (0.1 – 0.7)	**0.005**
	Stage [I (ref) vs II + III]	2.6 (1.3 – 5.3)	0.008
	Sex [M (ref) vs F]	0.7 (0.3 – 1.5)	0.380
	Age [≥65 (ref) vs <65]	1.4 (0.6 – 3.1)	0.391
TCF1+%	TCF1+% [L (ref) vs H]	2.0 (0.9 – 4.2)	0.064
	Stage [I (ref) vs II + III]	2.1 (1.1 – 4.3)	0.034
	Sex [M (ref) vs F]	0.7 (0.3 – 1.6)	0.420
	Age [≥65 (ref) vs <65]	1.2 (0.6 – 2.5)	0.650
TCF1+	TCF1+ [L (ref) vs H]	1.8 (0.9 – 3.5)	0.099
	Stage [I (ref) vs II + III]	2.2 (1.1 – 4.3)	0.027
	Sex [M (ref) vs F]	0.7 (0.3 – 1.6)	0.440
	Age [≥65 (ref) vs <65]	1.2 (0.6 – 2.6)	0.622
CD8+TCF1+/CD8+	CD8+TCF1+/CD8+ [L (ref) vs H]	1.8 (0.8 – 4.0)	0.163
	Stage [I (ref) vs II + III]	2.2 (1.1 – 4.3)	0.031
	Sex [M (ref) vs F]	0.7 (0.3 – 1.5)	0.393
	Age [≥65 (ref) vs <65]	1.3 (0.6 – 2.8)	0.514
CD4+	CD4+ [L (ref) vs H]	0.3 (0.1 – 0.7)	**0.005**
	Stage [I (ref) vs II + III]	2.2 (1.0 – 4.9)	0.060
	Sex [M (ref) vs F]	0.7 (0.3 – 1.6)	0.370
	Age [≥65 (ref) vs <65]	1.1 (0.5 – 2.7)	0.829
CD4+TCF1+	CD4+TCF1+ [L (ref) vs H]	0.4 (0.2 – 0.9)	**0.043**
	Stage [I (ref) vs II + III]	2.1 (0.9 – 4.7)	0.080
	Sex [M (ref) vs F]	0.7 (0.3 – 1.8)	0.521
	Age [≥65 (ref) vs <65]	1.3 (0.5 – 3.1)	0.596
CD79α+TCF1+/TCF1+	CD79α+TCF1+/TCF1+ [L (ref) vs H]	2.0 (0.8 – 5.2)	0.155
	Stage [I (ref) vs II + III]	2.5 (1.2 – 5.1)	0.010
	Sex [M (ref) vs F]	0.8 (0.4 – 1.7)	0.558
	Age [≥65 (ref) vs <65]	1.0 (0.5 – 2.3)	0.921
PD-L1	PD-L1 [No expr. (ref) vs Expr.]	0.4 (0.1 – 1.0)	0.057
	Stage [I (ref) vs II + III]	3.2 (1.3 – 7.6)	0.009
	Sex [M (ref) vs F]	0.5 (0.2 – 1.4)	0.198
	Age [≥65 (ref) vs <65]	2.0 (0.8 – 4.5)	0.116

Significant *P* values (< 0.05) linked with the biomarkers’ expression are written in bold.

## Discussion

The current study provides novel insights into the TME topography of NSCLC considering TCF1^+^ TILs, which is important not only from an immunophenotypic perspective but also for the clinical management of NSCLC patients. Understanding anti-tumor responses within the TME could help us to develop novel prognostic immune-associated biomarkers and allow us to better stratify and more appropriate use of immunotherapeutic approaches. Spatial profiling of TCF1^+^ immune cells and assessment of their clinical impact allowed several novel conclusions.

The immunophenotypic analysis of TCF1-expressing immune cells in the TME demonstrated that the vast majority of TCF1^+^ cells are CD79α^+^ B cells and CD4^+^ T cells, while CD8^+^ T cells comprise the minority in the TC (**Figures 3A, B**). Along this line, we demonstrated the presence of infiltrating plasma cells in the TME co-expressing TCF1 (**Figure 3C**). Bio-informatic analysis confirmed the expression of TCF1 mRNA (*TCF7*) in tumor infiltrating B cells and additionally demonstrated that TCF1^+^ B cells in NSCLC belong to memory/GC and naïve/transitional B cells (**Figure 2D**). To the best of our knowledge, this is the first study showing TCF1 immunopositivity in B cells, including plasma cells, in the TME of NSCLC. TCF1 expression in B cells and plasma cells was detected by single-cell transcriptomic analysis and independently confirmed by immunohistochemistry. Notably, protein-level TCF1 appeared higher than transcript levels, consistent with established evidence that mRNA abundance does not reliably predict protein expression due to extensive post-transcriptional, translational, and protein-stability regulation (25). Among CD4^+^TCF1^+^ cells, our analysis revealed CD4^+^ Treg and follicular helper T cells expressing TCF1 (**Figure 2D**). Consistently, it was recently demonstrated that CD4^+^ follicular helper T cells expressing TCF1 closely resemble stem-like progenitor exhausted CD8^+^ T cells as they maintain the capacity for proliferation and responsiveness to anti-PD-1 therapy (26). The same study demonstrated the presence of CD4^+^ T cells with low TCF1 expression and high lymphocyte-activation gene 3 (LAG3) levels, which have been reported in terminally differentiated exhausted CD8^+^ T cells (26). The findings draw parallels between CD4^+^ and CD8^+^ T cell exhaustion. Although CD4^+^ T cell exhaustion remains understudied, evidence suggests that these cells can experience exhaustion in cancer (27), underscoring the importance of assessing CD4^+^TCF1^+^ T cells in TME. Importantly, a significant percentage of TCF1-expressing immune cells were negative for CD8, CD4 and CD79α markers as detected by immunohistochemistry (**Figures 3A, B**), whereas single cell RNA-seq analyses confirmed that TCF1 is also expressed by myeloid and mast cells in NSCLC, opening a new dimension in TCF1^+^ landscape and warrants further in-depth investigation (**Figure 2**). Additionally, we observed TCF1 expression by cancer cells, also previously reported by our group in breast cancer patients (13) (**Figure 1D**). Notably, we found that increased TCF1(%) in the TC by cancer cells was associated with a dismal prognosis (**Table 3**). Overall, TCF1 expression by cancer cells is an understudied issue and warrants further investigation.

To capture the tumor microenvironment heterogeneity, we assessed the spatial distribution of TCF1-expressing T and B cells separately in the TC and the IF. Both CD8^+^TCF1^+^ and CD4^+^TCF1^+^ T cell counts were higher in the IF versus the TC (**Figures 1A**, **4A**), while no significant differences between the two regions were observed for CD79α^+^TCF1^+^ B cells (**Supplementary Figure S6**), providing an explanation why CD79α^+^ B cells represented the predominant TCF1^+^ expressing cells in the TC. Interestingly, PD1^+^TCF1^-^ cells overexceeded PD1^+^TCF1^+^ cells irrespective of tumor area (**Figure 5B**), supporting the notion that the predominant state of TILs in the NSCLC TME has features of terminally differentiated exhaustion. To the best of our knowledge, this is the first study assessing the spatial distribution separately of progenitor and terminally differentiated exhausted TILs in TME of NSCLC. Our findings are in line with a previous study showing that terminally exhausted T cells (defined as CD8^+^PD1^+^TCF1^-^) are the major subpopulation of exhausted T cells in head and neck carcinoma patients, although the impact of immune cell topography was not reported (28). Besides, we demonstrated that PD-L1 positivity is significantly associated with increased PD1^+^TCF1^-^ cell infiltrate irrespective of the tumor region, which could be the consequence of the immunoregulatory role of the PD-L1/PD-1 axis in T cell exhaustion (7).

The analysis of the prognostic impact of TCF1-expressing T and B cells separately in the TC and the IF revealed some interesting findings. We demonstrated that increased CD8^+^ T cell infiltrate in both the TC and the IF was associated with favorable DFS (**Figure 1B**, **Table 1**). Most studies acknowledge the significant association between high parenchymal CD8^+^ T cell density with favorable clinical outcome in NSCLC, although there are studies that do not support a statistically significant correlation (4). This discrepancy could be attributed to methodological reasons.

We further demonstrated that increased CD8^+^TCF1^+^ cell count in the TC but not in the IF is an independent prognostic marker significantly associated with worse DFS (**Tables 2**, **3**). A previous study by Wang et al. found that both high CD8^+^ and high CD8^+^TCF1^+^ T cells are significantly associated with improved DFS in lung adenocarcinoma patients (29). This discrepancy in the prognostic role of CD8^+^TCF1^+^ cell count could be attributed to methodological issues, since the study by Wang et al. did not consider spatial distribution of CD8^+^TCF1^+^ cells (29). Furthermore, the prognostic value of CD8^+^TCF1^+^ T cells seems to be even more complicated; we recently demonstrated that increased levels of CD8^+^TCF1^+^ T cell density is associated with improved DFS only in triple-negative but not in luminal type A invasive breast carcinoma patients (13). This finding underscores the importance of tumor context in determining the prognostic impact of CD8^+^TCF1^+^ cell density (13). It is interesting to note that both PD1^+^TCF1^+^ and PD1^+^TCF1^-^ cells have no impact on patients’ clinical outcome irrespective of their spatial distribution (**Supplementary Table 3**) as previously reported (10). Within this context, Førde et al. demonstrated the clinical relevance of high CD8^+^PD1^-^TCF1^+^ and CD8^-^ PD1^+^TCF1^+^ cell infiltration, which is associated with prolonged disease-specific survival in NSCLC patients (30). These discrepancies could be attributed to the heterogeneity of exhausted TILs as well as to methodological reasons. Regarding CD79α^+^ cell density, the survival analysis data revealed only a trend toward a better DFS in the TC but not in the IF (**Supplementary Table 2**) which is in line with a previous study showing improved prognostic impact of CD79α^+^CD20^+^ cell count in operable NSCLC patients (31).

Finally, increased CD4^+^ and CD4^+^TCF1^+^ cell count were independent prognostic markers significantly associated with improved DFS only in the IF but not in the TC (**Tables 2**, **3**). These findings gain further significance considering the abundance of TCF1-expressing CD79α^+^ B and CD4^+^ T cells in TME. Cumulatively, this data highlights the importance of considering immune cell topography when evaluating survival outcomes.

We also demonstrated that increased PD-L1 TPS, is an independent prognostic marker associated with improved DFS in the TC and the IF (**Tables 2**, **3**). Notably, increased PD-L1 expression was associated with increased CD8^+^ cells, which may explain the favorable prognostic value of PD-L1 status as already reported in our recent study on triple-negative breast carcinoma (13). In the literature, the prognostic value of PD-L1 in NSCLC is not clear. Some studies have reported that increased PD-L1 status (assessing both mRNA and protein levels) is associated with favorable clinical outcomes (32, 33), while others have found that increased PD-L1 status predicts poor prognosis (34, 35). A meta-analysis demonstrated that increased PD-L1 expression predicts poor survival in NSCLC patients; importantly, this association was not statistically significant after adjustment for postoperative chemotherapy or radiotherapy (36). Another meta-analysis assessed the prognostic value of PD-L1 transcription and found that high *PD-L1* expression was associated with a greater risk of recurrence in late-stage NSCLC patients, while it was associated with improved clinical outcomes in early-stage NSCLC patients (37). These disparities could be attributed to several factors, including the different experimental procedures, such as assessment protocols, use of different PD-L1 antibodies, and a lack of standardized methods.

Our study has some limitations. While double PD1/TCF1 immunostaining may serve as a valuable approach for assessing T cell exhaustion, our findings highlighting TCF1 expression across various T and B cell populations, underscore the need for a more comprehensive analysis. Specifically, simultaneous assessment of PD1/TCF1 expression alongside the characterization of distinct T and B cell subsets within the TME is warranted. Besides, the trial was conducted before the approved use of immunotherapy in the adjuvant/peri-operative setting, therefore immunotherapy response could not be assessed. Additionally, an overall survival analysis was not performed because there were too few events (e.g. deaths) within the study population to produce statistically reliable results. Future studies with larger patient cohorts are required to confirm and expand our findings.

## Conclusion

**Figure 7:**
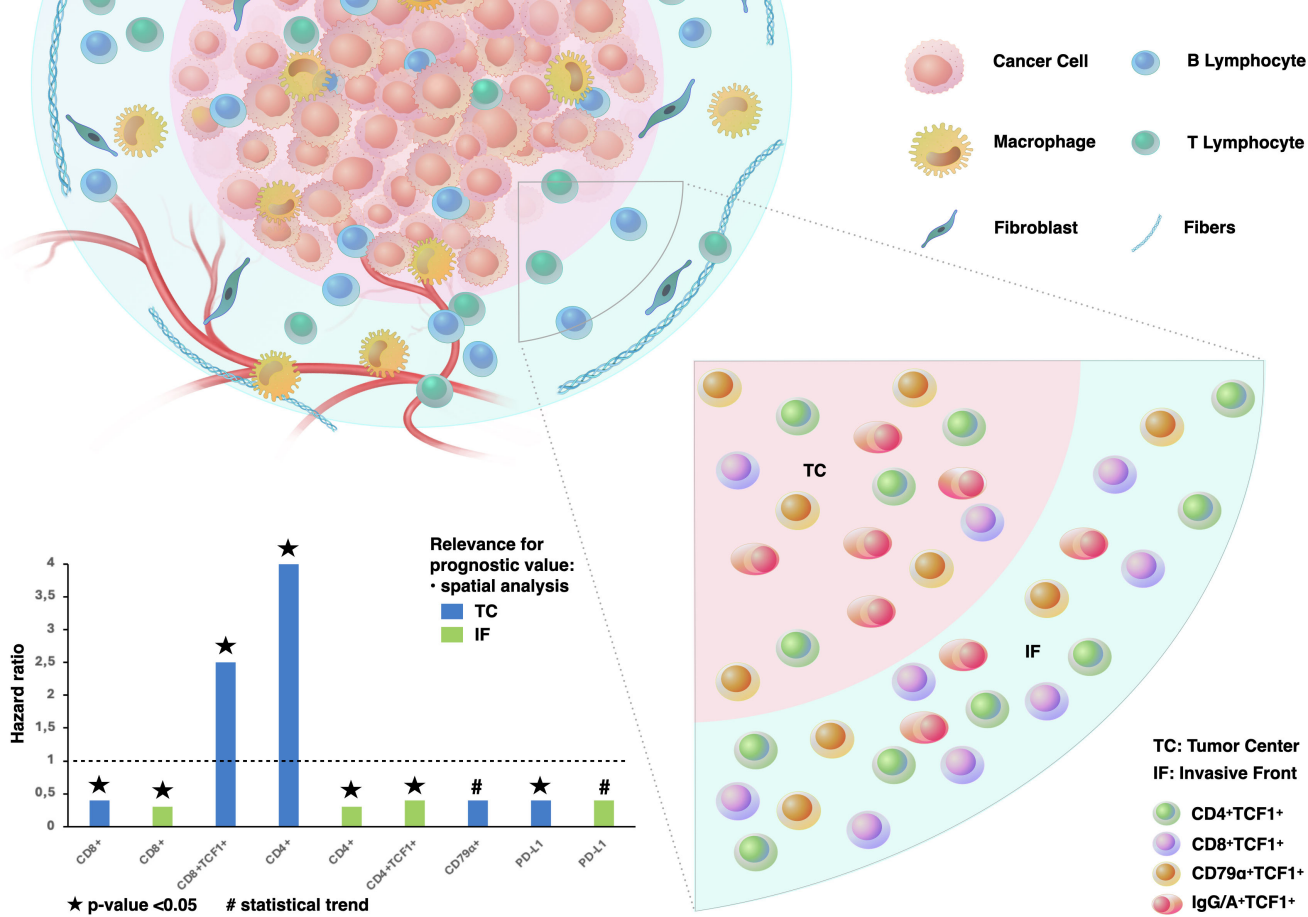
TCF1-expressing T and B cells in the lung TME. The vast majority of TCF1^+^ immune cells are CD79α^+^ B cells and CD4+ T cells, while CD8^+^ T cells comprise the minority of TCF1-expressing cells in non-small cell lung cancer (NSCLC). Independent prognostic impact of TCF1-expressing T and B cells on disease-free survival (as shown in multivariate Cox regression analyses), based on immune cell topography.

In summary, these findings contribute to the understanding of immune cell topography and clinical value of TCF1^+^ T and B cells in lung TME, providing a new perspective for assessing TILs in NSCLC. The abundance of CD79α^+^ B cells including plasma cells and CD4^+^ T cells in the TC, along with the distinct clinical value of CD8^+^TCF1^+^ and CD4^+^TCF1^+^ cells based on spatial distribution, opens a new dimension conceptually in the critical role of TCF1-expressing cells in tumor control (**Figure 7**). This, in turn, could aid in the development of more efficient biomarkers with prognostic and predictive value and improve patient stratification for cancer immunotherapy approaches.

## Data Availability

The original contributions presented in the study are included in the article/**Supplementary Material**. Further inquiries can be directed to the corresponding author.

## Supplementary material

Supplementary Figure 1 Clustering of single-cell RNA sequencing data reveals batch effects. **(A)** Elbow plot indicates how many principle components (PCs) should be used for downstream clustering of cells. The Seurat object was normalized by NormalizeData(). PCs 1:8 were selected for clustering. Clustering was performed with a resolution of 0.5. **(B)** Clusters colored by study accession ID reveals batch effects in the data.

Supplementary Figure 2 Batch correction using the RPCA integration method. **(A)** UMAP plot by study accession ID shows that batch effects have been corrected. The Seurat object was normalized by SCTransform() and clustered with a resolution of 0.1. **(B–D)** UMAP plots split by associated metadata including patient ID, stage of disease, and NSCLC subtype.

Supplementary Figure 3 Cell type annotation of single-cell clusters **(A)** UMAP plot showing cell type annotated Seurat clusters. **(B)** Dotplot demonstrates key markers used to annotated cell types including pan-B cell markers (CD19, MS4A1, CD79A, CD79B, CD27), T cell markers (CD2, CD3D, CD3E, CD3G, CD8A, CD8B), myeloid cell markers (CD14, CD68, CD163, CSF1R), plasma cell markers (CD38, SDC1, JCHAIN, MZB1), mast cell markers (KIT, GATA2, CPA3), ciliated cell markers (CAPS, TPPP3, C20orf85), and cancer-associated fibroblast (CAF) markers (DCN, COL1A1, COL1A2). **(C)** Dotplot demonstrates key markers used to annotated cell types including CD4+ T cell markers (CD4), CD8+ T cell markers (CD8A, CD8B), T regulatory cell markers (FOXP3), follicular helper T cell markers (PDCD1, TOX), plasma cell markers (CD38, SDC1, JCHAIN, MZB1), and pan-B cell markers (CD19, MS4A1, CD79A, CD79B, CD27) in combination with markers for naive/transitional B cells (MME, IGHM, TNFRSF13C, TCL1A, IL4R) and D. memory/GC B cells (BCL6, CXCR5, AICDA).

Supplementary Figure 4 Quantification of CD8^+^ cells in the tumor center (TC) and invasive front (IF). HPF, high power field.

Supplementary Figure 5 Quantification of CD4^+^ cells between the tumor center (TC) and the invasive front (IF). HPF, high power field.

Supplementary Figure 6 Quantification of CD79α^+^ and CD79α^+^TCF1^+^ cells between the tumor center (TC) and the invasive front (IF). HPF, high power field.

## Glossary

CI: confidence interval
DFS: Disease free survival
FFPE: formalin fixed paraffin embedded
HR: hazard ratio
ICIs: immune-checkpoint inhibitors
IF: invasive front
IHC: immunohistochemistry
LAG3: lymphocyte-activation gene 3
NSCLC: non-small cell lung carcinoma
PC: principal component
PD-1: programmed death 1
PD-L1: programmed death ligand 1
TC: tumor center
TCF1: T cell factor 1
TILs: Tumor infiltrating lymphocytes
TME: tumor microenvironment
TPS: Tumor proportion score
Treg: T regulatory cells

## References

[1] Siegel RL Miller KD Fuchs HE Jemal A . Cancer statistics, 2022. CA Cancer J Clin. (2022) 72:7–33. doi: 10.3322/caac.21708, PMID: 35020204

[2] Uramoto H Tanaka F . Recurrence after surgery in patients with NSCLC. Transl Lung Cancer Res. (2014) 3:242–9. doi: 10.3978/j.issn.2218-6751.2013.12.05, PMID: 25806307 PMC4367696

[3] Brambilla E Le Teuff G Marguet S Lantuejoul S Dunant A Graziano S . Prognostic effect of tumor lymphocytic infiltration in resectable non-small-cell lung cancer. J Clin Oncol. (2016) 34:1223–30. doi: 10.1200/JCO.2015.63.0970, PMID: 26834066 PMC4872323

[4] Fridman WH Zitvogel L Sautes-Fridman C Kroemer G . The immune contexture in cancer prognosis and treatment. Nat Rev Clin Oncol. (2017) 14:717–34. doi: 10.1038/nrclinonc.2017.101, PMID: 28741618

[5] Gataa I Mezquita L Rossoni C Auclin E Kossai M Aboubakar F . Tumour-infiltrating lymphocyte density is associated with favourable outcome in patients with advanced non-small cell lung cancer treated with immunotherapy. Eur J cancer. (2021) 145:221–9. doi: 10.1016/j.ejca.2020.10.017, PMID: 33516050

[6] Blank CU Haining WN Held W Hogan PG Kallies A Lugli E . Defining 'T cell exhaustion'. Nat Rev Immunol. (2019) 19:665–74. doi: 10.1038/s41577-019-0221-9, PMID: 31570879 PMC7286441

[7] Baessler A Vignali DAA . T cell exhaustion. Annu Rev Immunol. (2024) 42:179–206. doi: 10.1146/annurev-immunol-090222-110914, PMID: 38166256

[8] McLane LM Abdel-Hakeem MS Wherry EJ . CD8 T cell exhaustion during chronic viral infection and cancer. Annu Rev Immunol. (2019) 37:457–95. doi: 10.1146/annurev-immunol-041015-055318, PMID: 30676822

[9] Miller BC Sen DR Al Abosy R Bi K Virkud YV LaFleur MW . Subsets of exhausted CD8(+) T cells differentially mediate tumor control and respond to checkpoint blockade. Nat Immunol. (2019) 20:326–36. doi: 10.1038/s41590-019-0312-6, PMID: 30778252 PMC6673650

[10] Koh J Kim S Woo YD Song SG Yim J Han B . TCF1(+)PD-1(+) tumour-infiltrating lymphocytes predict a favorable response and prolonged survival after immune checkpoint inhibitor therapy for non-small-cell lung cancer. Eur J cancer. (2022) 174:10–20. doi: 10.1016/j.ejca.2022.07.004, PMID: 35970031

[11] Wang XQ Danenberg E Huang CS Egle D Callari M Bermejo B . Spatial predictors of immunotherapy response in triple-negative breast cancer. Nature. (2023) 621:868–76. doi: 10.1038/s41586-023-06498-3, PMID: 37674077 PMC10533410

[12] Fang X Wu G Hua J Zhao P Shan M Wang N . TCF-1(+) PD-1(+) CD8(+)T cells are associated with the response to PD-1 blockade in non-small cell lung cancer patients. J Cancer Res Clin Oncol. (2022) 148:2653–60. doi: 10.1007/s00432-021-03845-7, PMID: 34725738 PMC11800887

[13] Ntostoglou K Theodorou SDP Proctor T Nikas IP Awounvo S Sepsa A . Distinct profiles of proliferating CD8+/TCF1+ T cells and CD163+/PD-L1+ macrophages predict risk of relapse differently among treatment-naïve breast cancer subtypes. Cancer Immunol Immunother. (2024) 73:46. doi: 10.1007/s00262-024-03630-8, PMID: 38349444 PMC10864422

[14] Huang H Ge J Fang Z Wu S Jiang H Lang Y . Precursor exhausted CD8(+)T cells in colorectal cancer tissues associated with patient's survival and immunotherapy responsiveness. Front Immunol. (2024) 15:1362140. doi: 10.3389/fimmu.2024.1362140, PMID: 38510246 PMC10950923

[15] Laumont CM Nelson BH . B cells in the tumor microenvironment: Multi-faceted organizers, regulators, and effectors of anti-tumor immunity. Cancer Cell. (2023) 41:466–89. doi: 10.1016/j.ccell.2023.02.017, PMID: 36917951

[16] Leong TL Bryant VL . B cells in lung cancer-not just a bystander cell: a literature review. Transl Lung Cancer Res. (2021) 10:2830–41. doi: 10.21037/tlcr-20-788, PMID: 34295681 PMC8264333

[17] Papalampros A Vailas M Ntostoglou K Chiloeches ML Sakellariou S Chouliari NV . Unique spatial immune profiling in pancreatic ductal adenocarcinoma with enrichment of exhausted and senescent T cells and diffused CD47-SIRPalpha expression. Cancers. (2020) 12., PMID: 32645996 10.3390/cancers12071825 PMC7408661

[18] Zilionis R Engblom C Pfirschke C Savova V Zemmour D Saatcioglu HD . Single-cell transcriptomics of human and mouse lung cancers reveals conserved myeloid populations across individuals and species. Immunity. (2019) 50:1317–34.e10. doi: 10.1016/j.immuni.2019.03.009, PMID: 30979687 PMC6620049

[19] Kim N Kim HK Lee K Hong Y Cho JH Choi JW . Single-cell RNA sequencing demonstrates the molecular and cellular reprogramming of metastatic lung adenocarcinoma. Nat Commun. (2020) 11:2285. doi: 10.1038/s41467-020-16164-1, PMID: 32385277 PMC7210975

[20] Maroni G Bassal MA Krishnan I Fhu CW Savova V Zilionis R . Identification of a targetable KRAS-mutant epithelial population in non-small cell lung cancer. Commun Biol. (2021) 4:370. doi: 10.1038/s42003-021-01897-6, PMID: 33854168 PMC8046784

[21] Hanley CJ Waise S Ellis MJ Lopez MA Pun WY Taylor J . Single-cell analysis reveals prognostic fibroblast subpopulations linked to molecular and immunological subtypes of lung cancer. Nat Commun. (2023) 14:387. doi: 10.1038/s41467-023-35832-6, PMID: 36720863 PMC9889778

[22] Camp RL Dolled-Filhart M Rimm DL . X-tile: a new bio-informatics tool for biomarker assessment and outcome-based cut-point optimization. Clin Cancer Res. (2004) 10:7252–9. doi: 10.1158/1078-0432.CCR-04-0713, PMID: 15534099

[23] Chu PG Arber DA . CD79: a review. Appl Immunohistochem Mol Morphol. (2001) 9:97–106. doi: 10.1097/00129039-200106000-00001, PMID: 11396639

[24] Isaeva OI Sharonov GV Serebrovskaya EO Turchaninova MA Zaretsky AR Shugay M . Intratumoral immunoglobulin isotypes predict survival in lung adenocarcinoma subtypes. J Immunother Cancer. (2019) 7:279. doi: 10.1186/s40425-019-0747-1, PMID: 31665076 PMC6819482

[25] Liu Y Beyer A Aebersold R . On the dependency of cellular protein levels on mRNA abundance. Cell. (2016) 165:535–50. doi: 10.1016/j.cell.2016.03.014, PMID: 27104977

[26] Zhou W Kawashima S Ishino T Kawase K Ueda Y Yamashita K . Stem-like progenitor and terminally differentiated T(FH)-like CD4(+) T cell exhaustion in the tumor microenvironment. Cell Rep. (2024) 43:113797. doi: 10.1016/j.celrep.2024.113797, PMID: 38363680

[27] Miggelbrink AM Jackson JD Lorrey SJ Srinivasan ES Waibl-Polania J Wilkinson DS . CD4 T-cell exhaustion: does it exist and what are its roles in cancer? Clin Cancer Res. (2021) 27:5742–52. doi: 10.1158/1078-0432.CCR-21-0206, PMID: 34127507 PMC8563372

[28] Wang D Fang J Wen S Li Q Wang J Yang L . A comprehensive profile of TCF1(+) progenitor and TCF1(-) terminally exhausted PD-1(+)CD8(+) T cells in head and neck squamous cell carcinoma: implications for prognosis and immunotherapy. Int J Oral Sci. (2022) 14:8. doi: 10.1038/s41368-022-00160-w, PMID: 35153298 PMC8841504

[29] Wang Y Ma L Chen Y Yun W Yu J Meng X . Prognostic effect of TCF1+ CD8+ T cell and TOX+ CD8+ T cell infiltration in lung adenocarcinoma. Cancer Sci. (2024) 115:2184–95. doi: 10.1111/cas.16177, PMID: 38590234 PMC11247562

[30] Forde D Kilvaer T Pedersen MI Blix ES Urbarova I Paulsen EE . High density of TCF1+ stem-like tumor-infiltrating lymphocytes is associated with favorable disease-specific survival in NSCLC. Front Immunol. (2024) 15:1504220. doi: 10.3389/fimmu.2024.1504220, PMID: 39749327 PMC11693705

[31] El Hadad J Schreiner P Vavricka SR Greuter T . The genetics of inflammatory bowel disease. Mol Diagn Ther. (2024) 28:27–3. doi: 10.1007/s40291-023-00678-7, PMID: 37847439 PMC10787003

[32] Velcheti V Schalper KA Carvajal DE Anagnostou VK Syrigos KN Sznol M . Programmed death ligand-1 expression in non-small cell lung cancer. Lab Invest. (2014) 94:107–16. doi: 10.1038/labinvest.2013.130, PMID: 24217091 PMC6125250

[33] Yang CY Lin MW Chang YL Wu CT Yang PC . Programmed cell death-ligand 1 expression in surgically resected stage I pulmonary adenocarcinoma and its correlation with driver mutations and clinical outcomes. Eur J cancer. (2014) 50:1361–9. doi: 10.1016/j.ejca.2014.01.018, PMID: 24548766

[34] Zhang Y Wang L Li Y Pan Y Wang R Hu H . Protein expression of programmed death 1 ligand 1 and ligand 2 independently predict poor prognosis in surgically resected lung adenocarcinoma. Onco Targets Ther. (2014) 7:567–73. doi: 10.2147/OTT.S59959, PMID: 24748806 PMC3990506

[35] Chen YB Mu CY Huang JA . Clinical significance of programmed death-1 ligand-1 expression in patients with non-small cell lung cancer: a 5-year-follow-up study. Tumori. (2012) 98:751–5. doi: 10.1177/030089161209800612, PMID: 23389362

[36] Sun JM Zhou W Choi YL Choi SJ Kim SE Wang Z . Prognostic significance of PD-L1 in patients with non-small cell lung cancer: A large cohort study of surgically resected cases. J Thorac Oncol. (2016) 11:1003–11. doi: 10.1016/j.jtho.2016.04.007, PMID: 27103510

[37] Chang CH Shih AC Chang YH Chen HY Chao YT Hsu YC . The prognostic significance of PD1 and PDL1 gene expression in lung cancer: A meta-analysis. Front Oncol. (2021) 11:759497. doi: 10.3389/fonc.2021.759497, PMID: 34868974 PMC8639141

