# Supplementary material for: Immunophenotyping TCF1-expressing TILs: spatial profiling and prognostic value in operable non-small cell lung cancer
Source: Front Immunol. 2026 Jan 22;17:1731337. doi: 10.3389/fimmu.2026.1731337 (PMC12872492; doi:10.3389/fimmu.2026.1731337)
Supplement: Supplementary Figure 1 — Clustering of single-cell RNA sequencing data reveals batch effects. (A) Elbow plot indicates how many principle components (PCs) should be used for downstream clustering of cells. The Seurat object was normalized by NormalizeData(). PCs 1:8 were selected for clustering. Clustering was performed with a resolution of 0.5. (B) Clusters colored by study accession ID reveals batch effects in the data. [file DataSheet1.zip › Supplementary Tables.docx]

**Supplementary Table 1:** Clinicopathological data of patient cohort

| **N=102** | **No (%)** |
| --- | --- |
| **Age (years)** |  |
| Median (min – max) | 68.0 (39 – 86) |
| **Sex** |  |
| Men | 67 (65.7) |
| Women | 35 (34.3) |
| **Smoking status** |  |
| Active smoker | 53 (52.0) |
| Ex – smoker | 34 (33.3) |
| Never – smoker | 3 (2.9) |
| N/A | 12 (11.8) |
| **Histology** |  |
| SCC | 46 (45.1) |
| ADC | 49 (48.0) |
| Others**^*^** | 7 (6.9) |
| **Stage at diagnosis (pTNM, 8^th^)** |  |
| IA | 29 (28.4) |
| IB | 27 (26.5) |
| IIA | 4 (3.9) |
| IIB | 19 (18.6) |
| IIIA | 23 (22.5) |
| **Adjuvant Chemotherapy** |  |
| Yes # | 52 (51.0) |
| No | 50 (49.0) |
| **Median Follow up (mo)** | 50.3 (6.6 – 73.5) |

**Note**: Others*: pleomorphic carcinoma and large cell carcinoma. Abbreviations: ADC: Adenocarcinoma; N/A: Non available; pTNM: pathological Tumor Node Metastasis; SCC: Squamous cell carcinoma. # platinum-based doublets without immunotherapy were used since the trial was conducted before the approved use of immunotherapy in the adjuvant/peri-operative setting.

**Supplementary Table 2**: KM and Univariate Cox regression analysis for DFS, evaluating the prognostic role of CD79α/TCF1 status separately in the tumor center (TC) and invasive front (IF)

|  | **DFS – TC** | | | | **DFS – IF** | | | |
| --- | --- | --- | --- | --- | --- | --- | --- | --- |
|  | **KM analysis** | | **Univ. Cox regression** | | **KM analysis** | | **Univ. Cox regression** | |
| **CD79α+** | **Median (range)** | **p-value** | **HR (95%C.I)** | **p-value** | **Median (range)** | **p-value** | **HR (95%C.I)** | **p-value** |
| Low (ref) | 17.0 (4.9 – 68.4) | 0.052 | 0.4 (0.2-1.0) | 0.058 | NR (2.9 – 68.3) | 0.118 | 0.6 (0.3-1.2) | 0.122 |
| High | NR (2.9 – 73.5) |  |  |  | NR (6.8 – 73.5) |  |  |  |
| **CD79α+TCF1+** |  |  |  |  |  |  |  |  |
| Low (ref) | NR (2.9 – 68.4) | 0.122 | 2.0 (0.8-4.8) | 0.129 | NR (2.9 – 68.4) | 0.176 | 1.7 (0.8-3.5) | 0.181 |
| High | 34.9 (6.8 – 73.5) |  |  |  | 40.0 (6.6 – 73.5) |  |  |  |
| **CD79α+TCF1+/TCF1+** |  |  |  |  |  |  |  |  |
| Low (ref) | NR (2.9 – 61.7) | 0.166 | 1.9 (0.7-5.0) | 0.173 | NR (2.9 – 73.5) | 0.061 | 1.9 (0.9-3.9) | 0.065 |
| High | NR (6.6 – 73.5) |  |  |  | 35.8 (6.6 – 67.7) |  |  |  |
| **CD79α+TCF1+/CD79α+** |  |  |  |  |  |  |  |  |
| Low (ref) | NR (2.9 – 68.4) | 0.165 | 0.5 (0.2-1.3) | 0.172 | NR (2.9 – 61.0) | 0.148 | 2.0 (0.8-5.2) | 0.156 |
| High | NR (7.8 – 73.5) |  |  |  | 44.2 (6.6 – 73.5) |  |  |  |

**Note**: ΝR: not reached

**Supplementary Table 3**: KM and Univariate Cox regression analysis for DFS evaluating the prognostic role of PD1/TCF1 status separately in the tumor center (TC) and invasive front (IF)

|  | **DFS – TC** | | | | **DFS – IF** | | | | |
| --- | --- | --- | --- | --- | --- | --- | --- | --- | --- |
|  | **KM analysis** | | **Univ. Cox regression** | | **KM analysis** | | | **Univ. Cox regression** | |
| **PD1+TCF1-** | **Median (range)** | **p-value** | **HR (95%C.I)** | **p-value** | **Median (range** | **p-value** | **HR (95%C.I)** | | **p-value** |
| Low (ref) | NR (2.9 – 73.5) | 0.121 | 0.5 (0.2-1.2) | 0.128 | NR (92.9 – 73.5) | 0.324 | 1.5 (0.7-3.5) | | 0.328 |
| High | NR (6.8 – 66.5) |  |  |  | 36.8 (6.8 – 62.3) |  |  |  |  |
| **PD1+TCF1+** |  |  |  |  |  |  |  | |  |
| Low (ref) | NR (2.9 – 73.5) | 0.522 | 1.3 (0.6-2.5) | 0.523 | NR (2.9 – 73.5) | 0.122 | 2.0 (0.8-4.8) | | 0.130 |
| High | NR (6.6 – 66.5) |  |  |  | 36.8 (7.2 – 62.3) |  |  |  |  |

**Note**: ΝR: not reached

**Supplementary Table 4:** CD8/TCF1 value assessment based on PD-L1 expression separately in the tumor center (TC) and invasive front (IF) groups in NSCLC patients

| **PD-L1 (TC)** | | | | | |
| --- | --- | --- | --- | --- | --- |
|  | **Mean ± SD** | | **Median (Q1-Q3)** | |  |
| **Biomarkers** | **No expression** | **Expression** | **No expression** | **Expression** | ***p*-value** |
| **CD8+** | 21.5 ± 14.9 | 38.5 ± 26.5 | 20.0 (12.5 – 24.7) | 30.3 (19.5 – 52.3) | **0.001** |
| **TCF1+** | 27.6 ± 20.8 | 34.9 ± 30.4 | 22.3 (14.2 – 37.3) | 23.1 (14.5 – 44.7) | 0.457 |
| **TCF1+% *** | 29.8 ± 30.4 | 25.4 ± 31.3 | 20.0 (2.0 – 60.0) | 13.5 (0 – 40.0) | 0.408 |
| **CD8+TCF1+** | 7.8 ± 9.8 | 8.6 ± 11.1 | 4.5 (2.5 – 9.7) | 4.7 (2.0 – 10.0) | 0.925 |
| **CD8+TCF1+/CD8+** | 0.3 ± 0.2 | 0.2 ± 0.2 | 0.3 (0.1 – 0.5) | 0.2 (0.1 – 0.4) | **0.024** |
| **CD8+TCF1+/TCF1+** | 0.3 ± 0.2 | 0.2 ± 0.2 | 0.3 (0.1 – 0.5) | 0.2 (0.1 – 0.4) | 0.271 |
| **PD-L1 (IF)** | | | | | |
|  | **Mean ± SD** | | **Median (Q1-Q3)** | |  |
| **Biomarkers** | **No expression** | **Expression** | **No expression** | **Expression** | ***p*-value** |
| **CD8+** | 25.0 ± 19.0 | 41.9 ± 26.5 | 22.0 (12.5 – 36.3) | 34.6 (26.7 – 54.5) | **0.001** |
| **TCF1+** | 38.9 ± 28.7 | 57.2 ± 22.1 | 28.0 (16.4 – 56.0) | 56.7 (43.7 – 69.0) | **0.002** |
| **TCF1+% *** | 29.0 ± 30.9 | 26.7 ± 29.4 | 20.0 (2.5 – 57.5) | 20.0 (5.0 – 40.0) | 0.887 |
| **CD8+TCF1+** | 11.3 ± 14.7 | 18.2 ± 16.9 | 5.9 (3.1 – 14.9) | 13.5 (8.1 – 22.0) | **0.006** |
| **CD8+TCF1+/CD8+** | 0.4 ± 0.2 | 0.4 ± 0.2 | 0.3 (0.2 – 0.5) | 0.4 (0.3 – 0.5) | 0.659 |
| **CD8+TCF1+/TCF1+** | 0.4 ± 0.3 | 0.3 ± 0.3 | 0.4 (0.1 – 0.5) | 0.3 (0.1 – 0.4) | 0.605 |

**Note**: Q1-Q3: Quartile 1 - 3 TCF1+(%) * refers to TCF1-expression by cancer cells. No Expression: TPS score <1; PD-L1 Expression: TPS score ≥1.

**Supplementary Table 5:** PD1/TCF1 values assessment based on PD-L1 expression groups separately in the tumor center (TC) and invasive front (IF) in NSCLC patients

| **PD-L1 (TC)** | | | | | |
| --- | --- | --- | --- | --- | --- |
|  | **Mean ± SD** | | **Median (Q1-Q3)** | |  |
| **Biomarkers** | **No expression** | **Expression** | **No expression** | **Expression** | ***p*-value** |
| **PD1+TCF1-** | 6.5 ± 7.9 | 13.8 ± 9.9 | 3.8 (0.9 – 8.8) | 13.9 (4.3 – 23.2) | **0.019** |
| **PD1+TCF1+** | 0.8 ± 1.1 | 1.2 ± 0.9 | 0.3 (0 – 1.1) | 1.0 (0.5 – 2.2) | 0.118 |
| **PD-L1 (IF)** | | | | | |
|  | **Mean ± SD** | | **Median (Q1-Q3)** | |  |
| **Biomarkers** | **No expression** | **Expression** | **No expression** | **Expression** | ***p*-value** |
| **PD1+TCF1-** | 7.7 ± 8.3 | 16.2 ± 12.6 | 5.5 (2.1 – 10.2) | 12.4 (5.1 – 26.6) | **0.021** |
| **PD1+TCF1+** | 2.6 ± 3.2 | 4.6 ± 4.6 | 1.3 (0.5 – 3.9) | 4.0 (1.6 – 5.7) | 0.056 |

**Note**: Q1-Q3: Quartile 1 - 3. No Expression: TPS score <1; PD-L1 Expression: TPS score ≥1.

**Supplementary Table 6**: KM analysis and Univariate Cox regression analysis for DFS evaluating the prognostic role of PD-L1 TPS separately in the tumor center (TC) and invasive front (IF).

|  | **DFS – TC** | | | | **DFS - IF** | | | |
| --- | --- | --- | --- | --- | --- | --- | --- | --- |
|  | **KM analysis** | | **Univ. Cox regression** | | **KM analysis** | | **Univ. Cox regression** | |
| **PD-L1** | **Median (range)** | **p-value** | **HR (95%C.I)** | **p-value** | **Median (range)** | **p-value** | **HR (95%C.I)** | **p-value** |
| No expression (ref) | NR (2.9 – 73.5) | 0.033 | 0.4 (0.2-0.9) | **0.039** | NR (2.9 – 68.4) | 0.037 | 0.4 (0.1-0.9) | **0.045** |
| Expression | NR (8.8 – 66.5) |  |  |  | NR (8.8 – 67.7) |  |  |  |

**Note**: ΝR: not reached. TPS: Tumor Proportion Score; No Expression: TPS score <1; PD-L1 Expression: TPS score ≥1.
