# Supplementary material for: Immunophenotyping TCF1-expressing TILs: spatial profiling and prognostic value in operable non-small cell lung cancer
Source: Front Immunol. 2026 Jan 22;17:1731337. doi: 10.3389/fimmu.2026.1731337 (PMC12872492; doi:10.3389/fimmu.2026.1731337)
Supplement: Supplementary Figure 1 — Clustering of single-cell RNA sequencing data reveals batch effects. (A) Elbow plot indicates how many principle components (PCs) should be used for downstream clustering of cells. The Seurat object was normalized by NormalizeData(). PCs 1:8 were selected for clustering. Clustering was performed with a resolution of 0.5. (B) Clusters colored by study accession ID reveals batch effects in the data. [file DataSheet1.zip › Supplementary Figures.pptx]

## Slide 1
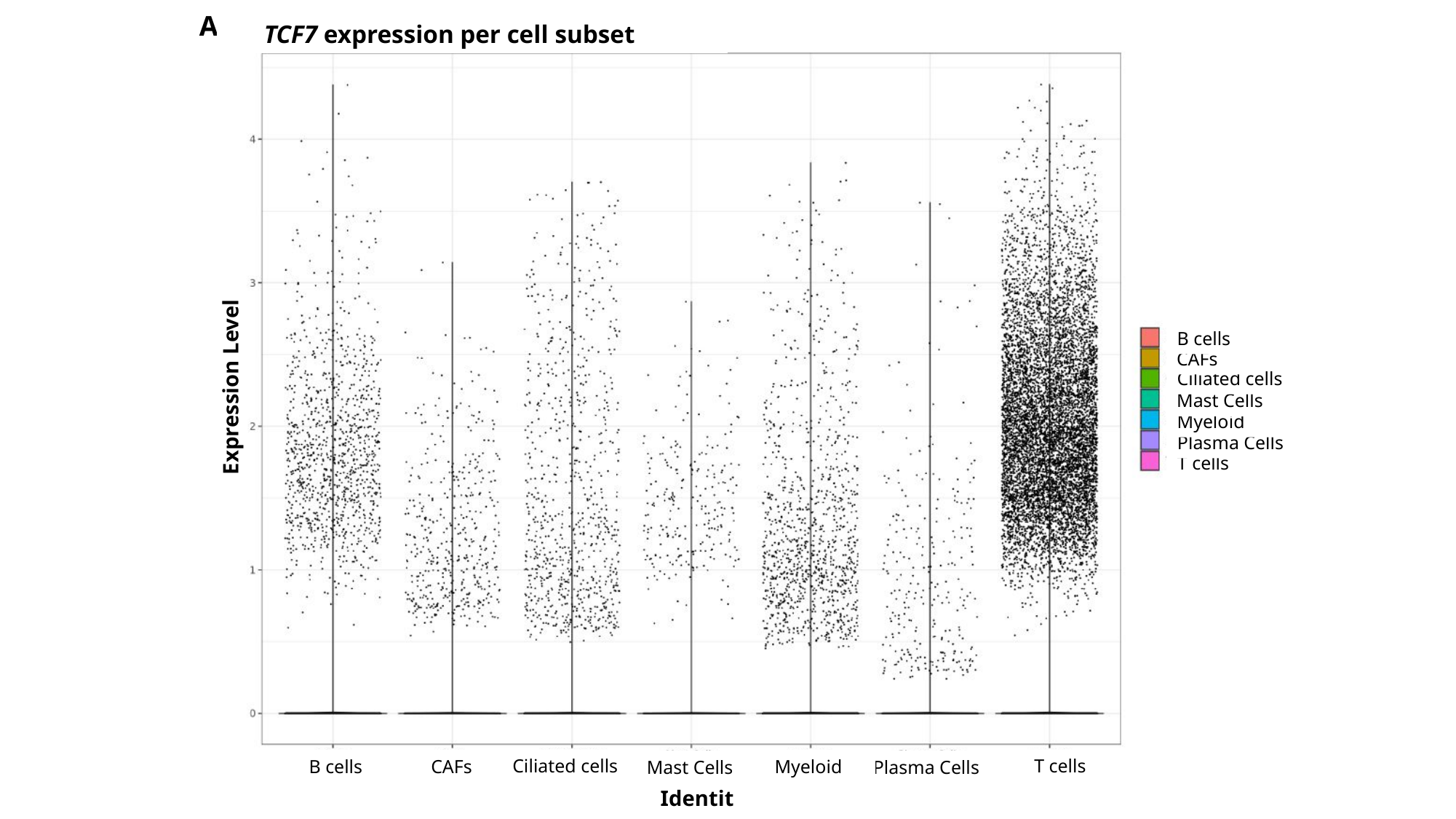

A
TCF7 expression per cell subset
Ciliated cells
T cells
Myeloid
B cells
CAFs
Mast Cells
Plasma Cells
B cells
CAFs
Ciliated cells
Expression Level
Mast Cells
Myeloid
Plasma Cells
T cells
Identity

## Slide 2
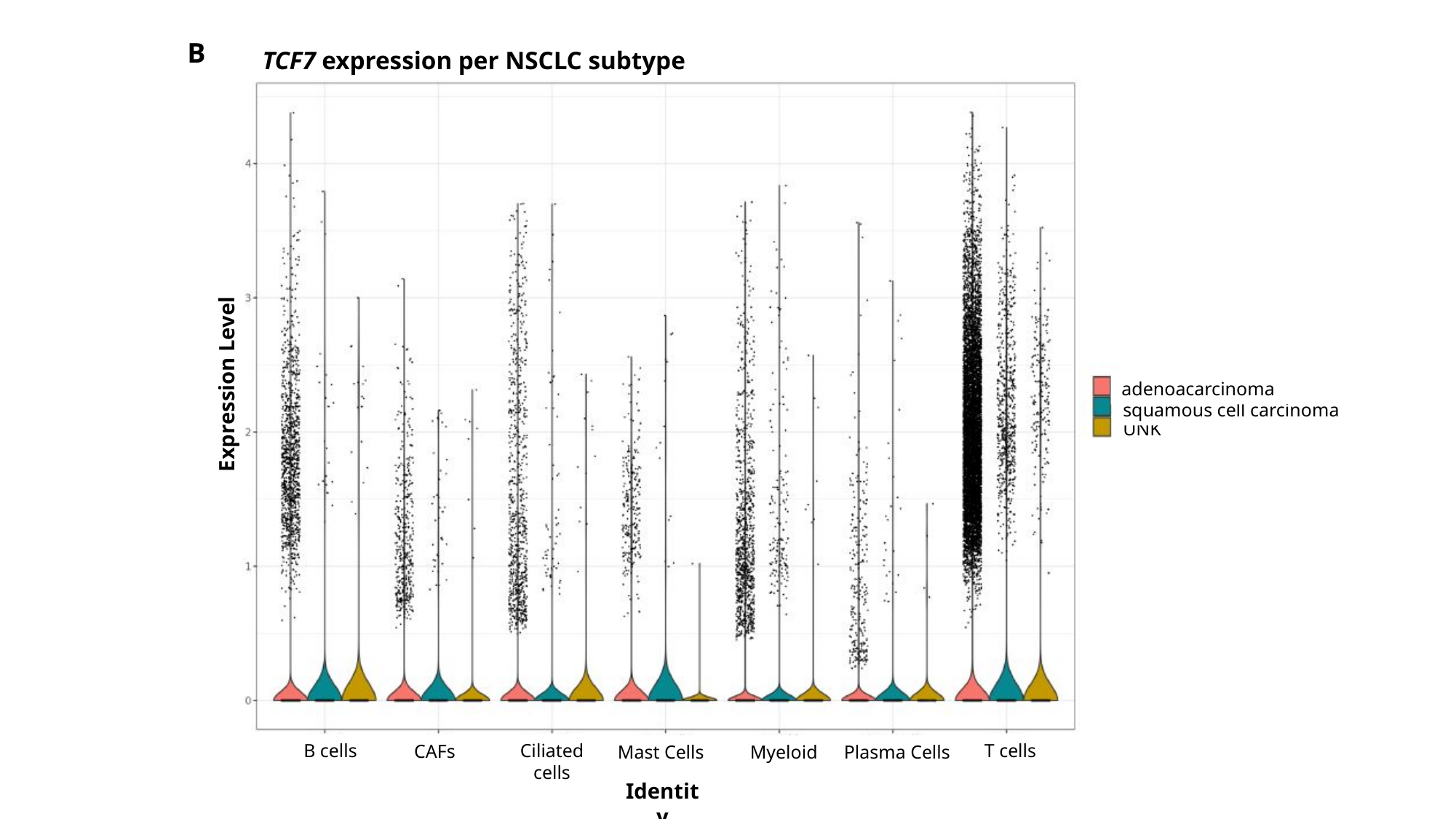

B
TCF7 expression per NSCLC subtype
Expression Level
adenoacarcinoma
squamous cell carcinoma
UNK
T cells
Ciliated cells
B cells
CAFs
Plasma Cells
Mast Cells
Myeloid
Identity

## Slide 3
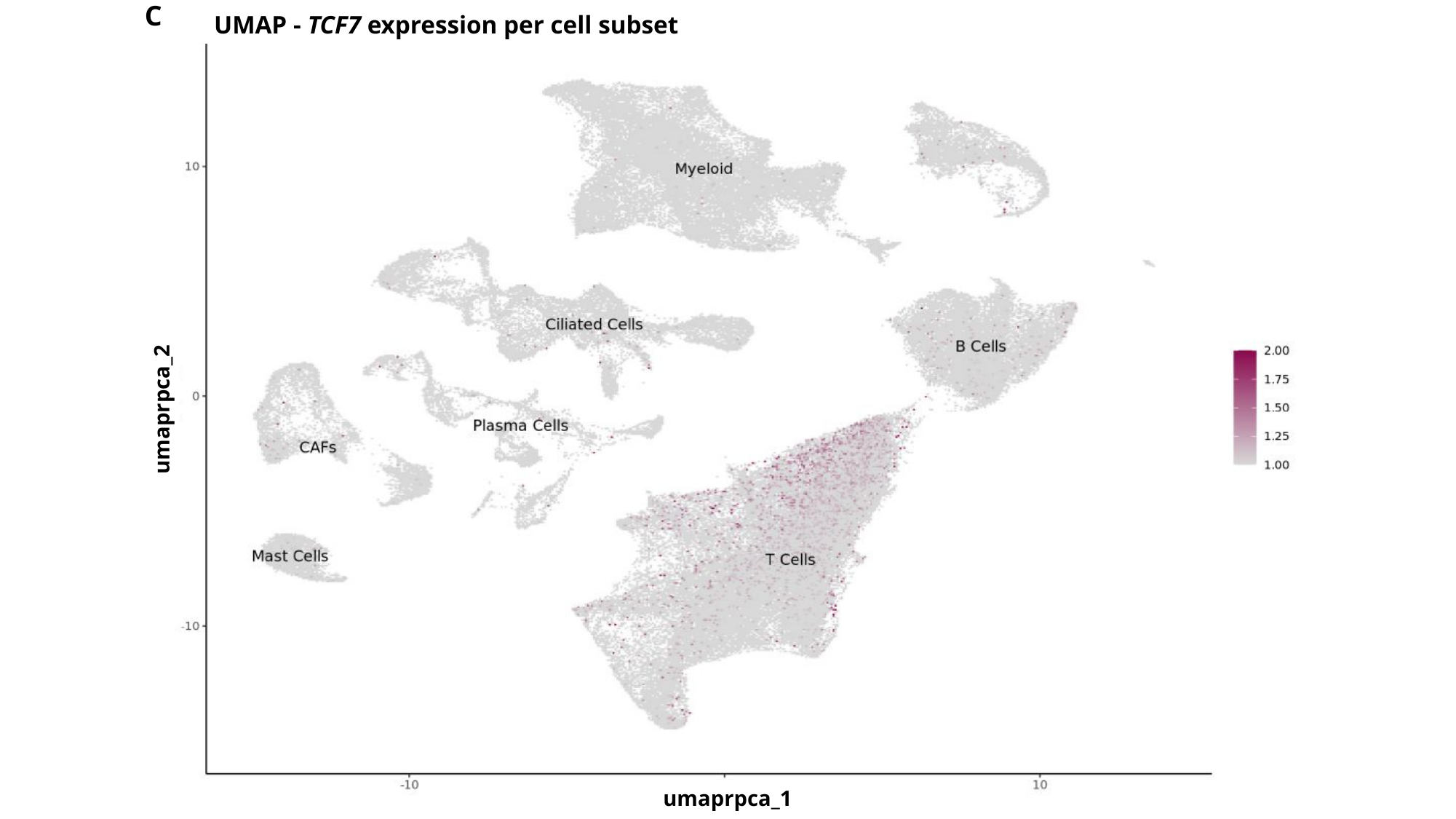

C
UMAP - TCF7 expression per cell subset
umaprpca_2
umaprpca_1

## Slide 4
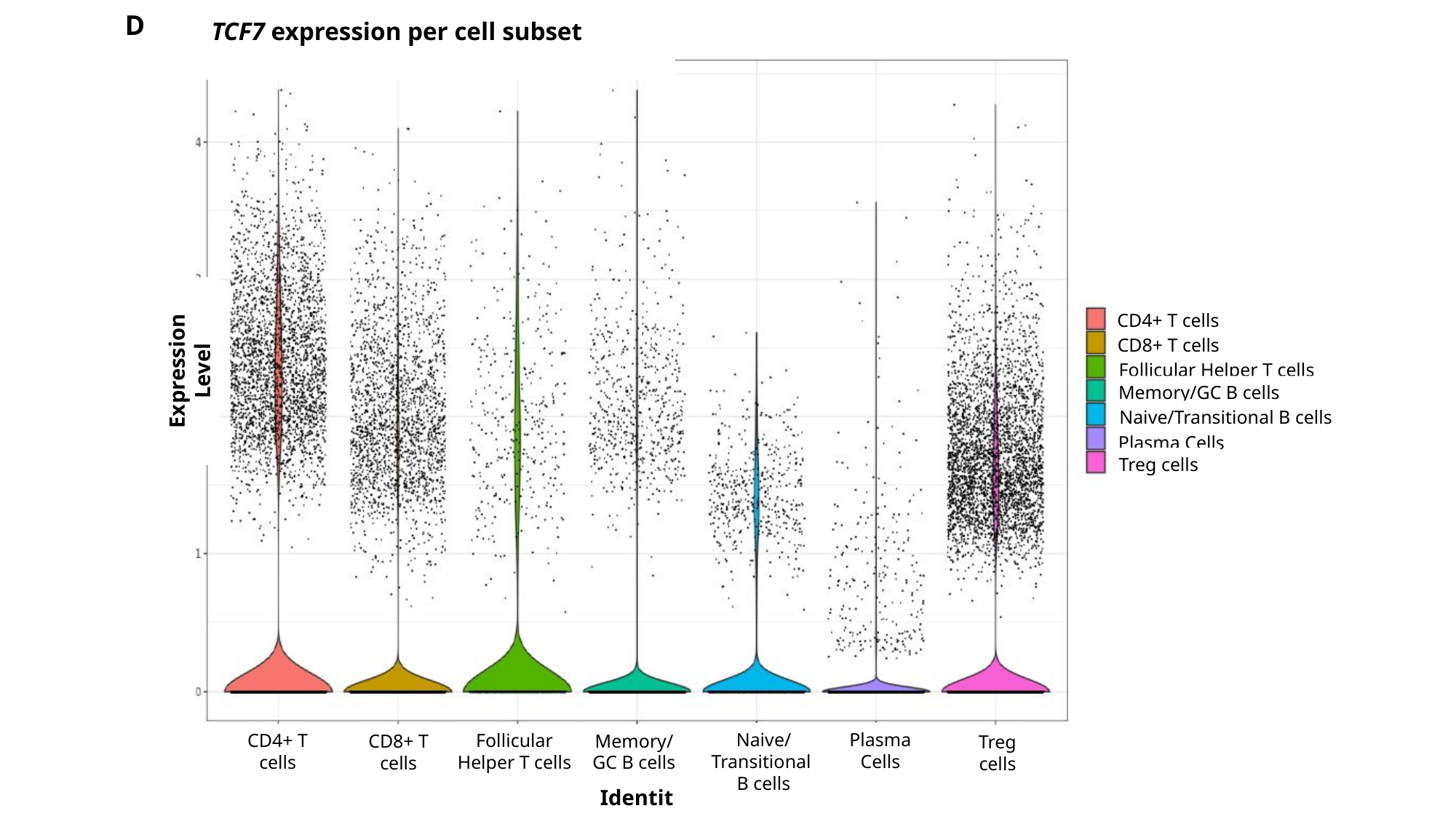

D
TCF7 expression per cell subset
CD4+ T cells
CD8+ T cells
Expression Level
Follicular Helper T cells
Memory/GC B cells
Naive/Transitional B cells
Plasma Cells
Treg cells
Plasma Cells
Naive/Transitional
B cells
CD4+ T cells
Follicular Helper T cells
Memory/GC B cells
CD8+ T cells
Treg cells
Identity
