# Supplementary material for: Immunophenotyping TCF1-expressing TILs: spatial profiling and prognostic value in operable non-small cell lung cancer
Source: Front Immunol. 2026 Jan 22;17:1731337. doi: 10.3389/fimmu.2026.1731337 (PMC12872492; doi:10.3389/fimmu.2026.1731337)
Supplement: Supplementary Figure 1 — Clustering of single-cell RNA sequencing data reveals batch effects. (A) Elbow plot indicates how many principle components (PCs) should be used for downstream clustering of cells. The Seurat object was normalized by NormalizeData(). PCs 1:8 were selected for clustering. Clustering was performed with a resolution of 0.5. (B) Clusters colored by study accession ID reveals batch effects in the data. [file DataSheet1.zip › Supplementary material.DOCX]

**Supplementary Material**

Optimal cut-off values for the tested markers in the tumor center (TC) and invasive front (IF)

| **Biomarker** | **TC** | **IF** |
| --- | --- | --- |
| CD8+ | 9.0 | 12.0 |
| TCF1% | 1.7% | 5% |
| TCF1+ | 40.0 | 63.0 |
| CD8+TCF1+ | 2.3 | 3.7 |
| CD8+TCF1+/CD8+ | 0.5 | 0.6 |
| CD8+TCF1+/TCF1+ | 0.1 | 0.5 |
| CD4+ | 50.3 | 26.5 |
| CD4+TCF1+ | 11.8 | 12.5 |
| CD4+TCF1+/TCF1+ | 0.2 | 0.4 |
| CD4+TCF1+/CD4+ | 0.2 | 0.4 |
| CD79+ | 22.0 | 80.0 |
| CD79+TCF1+ | 101.5 | 58.0 |
| CD79+TCF1+/TCF1+ | 0.5 | 0.7 |
| CD79+TCF1+/CD79+ | 0.8 | 0.3 |
| PD1+TCF1- | 12.7 | 17.5 |
| PD1+TCF1+ | 1.5 | 6.8 |

**X-tile graphs**

**Tumor center (TC)**

**
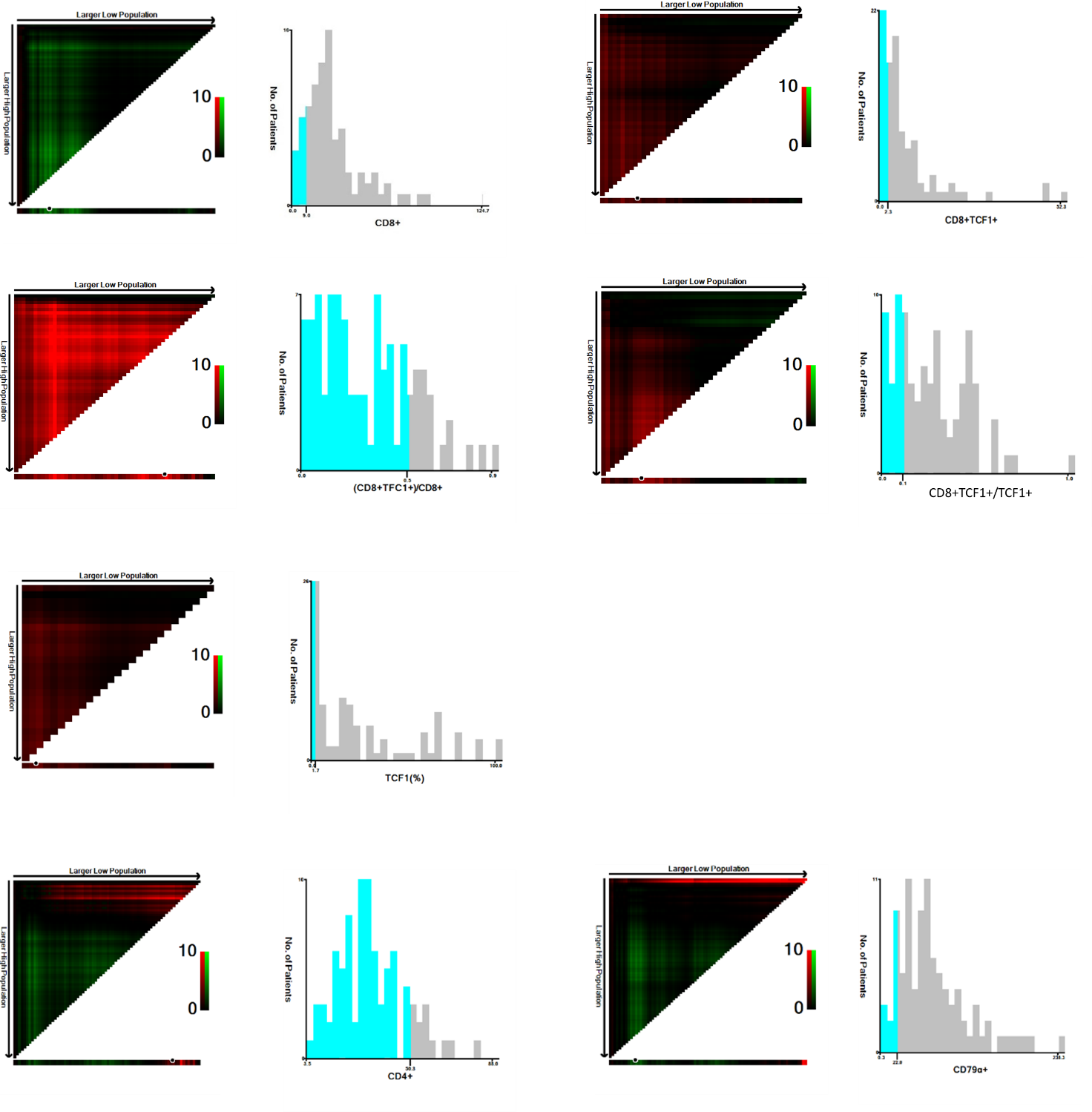
**

**Invasive front (IF)**

**
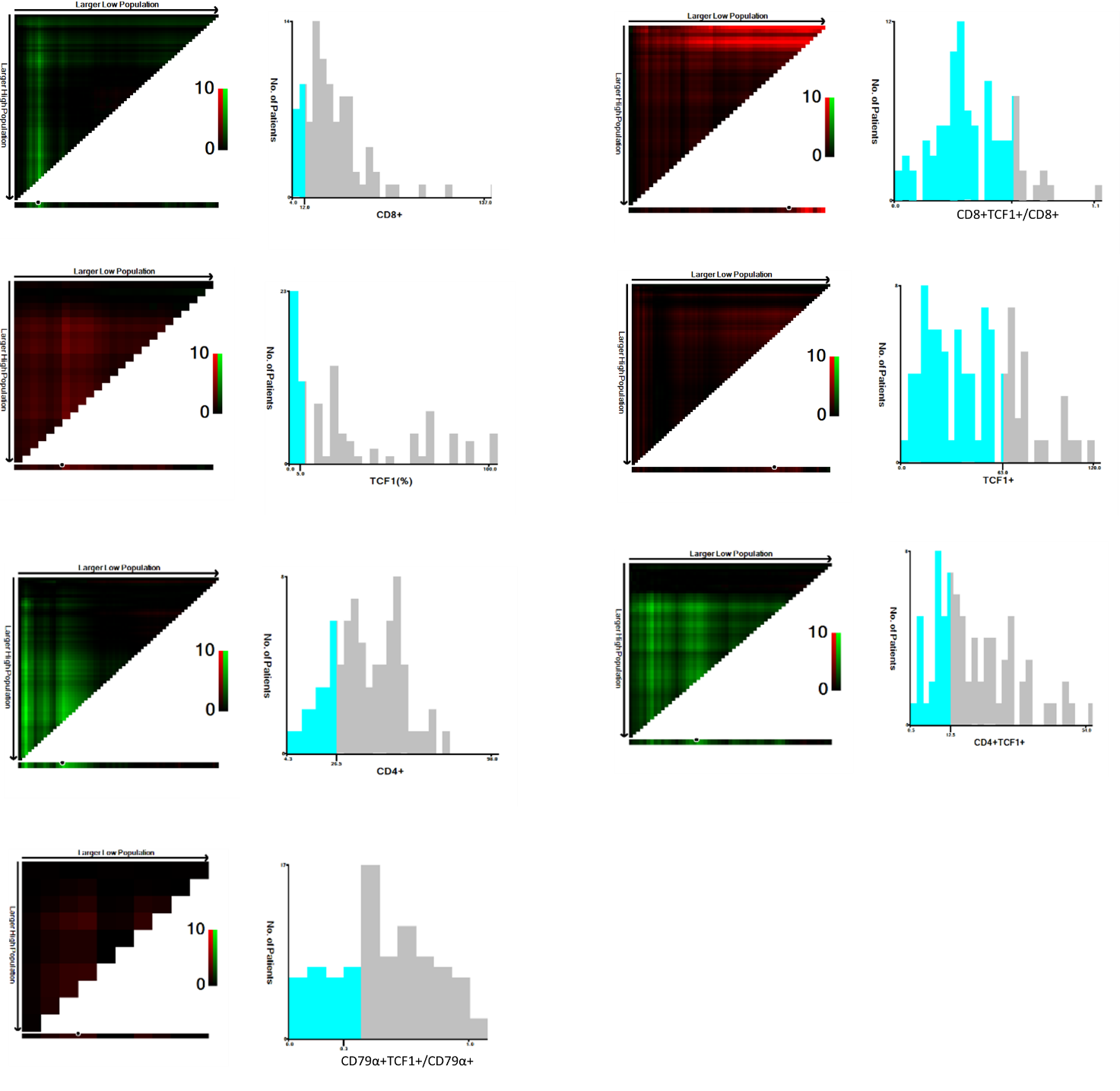
**
